# Supplementary material for: TAPBPR promotes antigen loading on MHC-I molecules using a peptide trap
Source: Nat Commun. 2021 May 26;12:3174. doi: 10.1038/s41467-021-23225-6 (PMC8154891; doi:10.1038/s41467-021-23225-6)
Supplement: Supplementary file 1 — Supplementary Information [file 41467_2021_23225_MOESM1_ESM.pdf]

Supplementary Information

TAPBPR Promotes Antigen Loading on MHC-I Molecules Using a Peptide Trap

Andrew C. McShan[1†], Christine A. Devlin[2†], Giora I. Morozov[1], Sarah A. Overall[3], Danai Moschidi[3], Neha Akella[2], Erik Procko[2*] and Nikolaos G. Sgourakis[1,4*]

[1]Center for Computational and Genomic Medicine, Department of Pathology and Laboratory Medicine, The Children's Hospital of Philadelphia, 3401 Civic Center Blvd, Philadelphia, PA, 19104, USA

[2]Department of Biochemistry and Cancer Center at Illinois, University of Illinois, Urbana, IL 61801, USA.

[3]Department of Chemistry and Biochemistry, University of California Santa Cruz, Santa Cruz, CA 95064, USA.

[4]Department of Biochemistry and Biophysics, Perelman School of Medicine, University of Pennsylvania, 3400 Civic Center Blvd, Philadelphia, PA, 19104, USA

† These authors contributed equally to this work.

* To whom correspondence should be addressed: Erik Procko (procko@illinois.edu) or Nikolaos G. Sgourakis (Nikolaos.Sgourakis@Pennmedicine.upenn.edu).

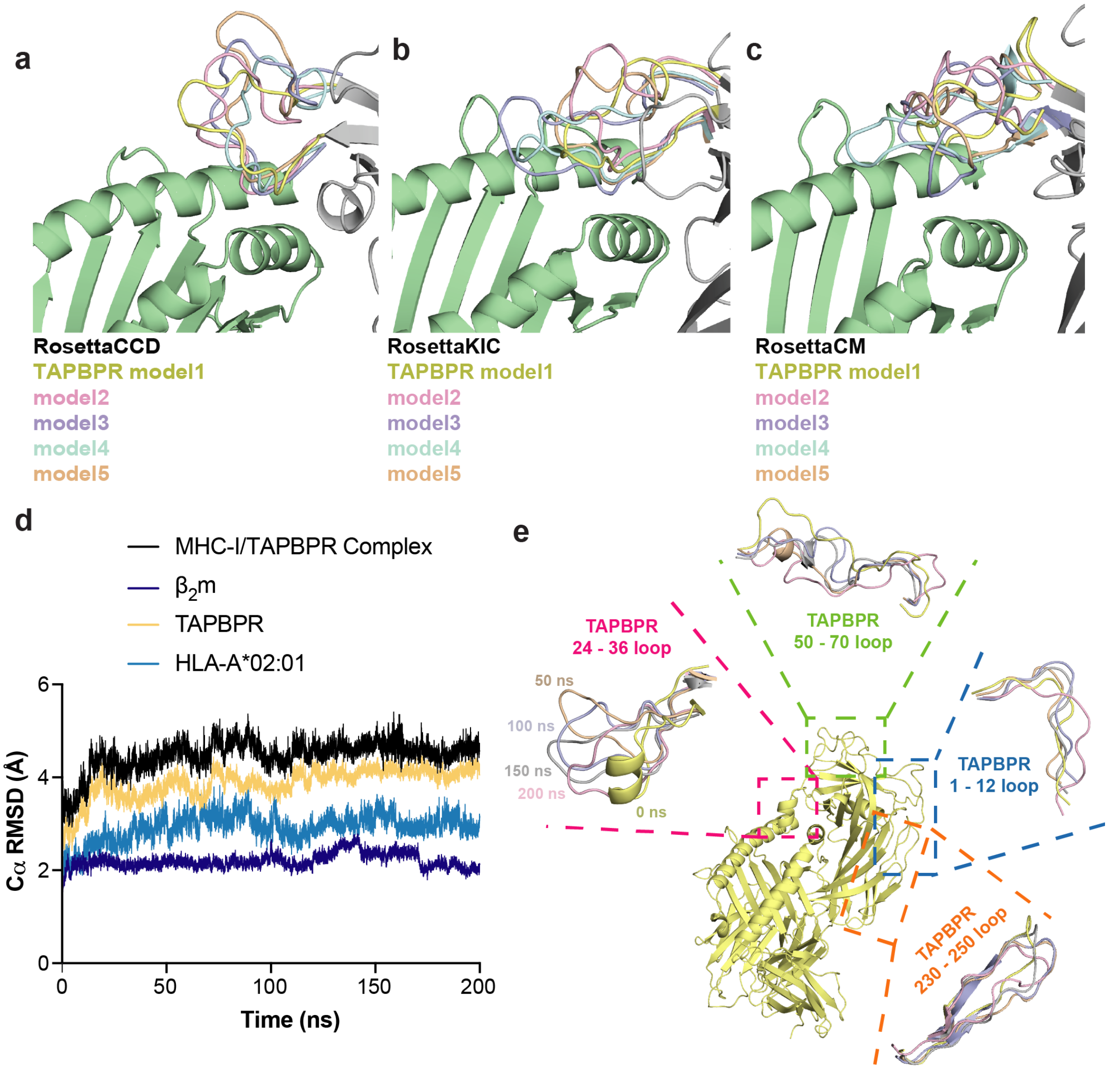

Supplementary Fig. 1. Conformational variation of the TAPBPR G24-R36 loop. (a-c) Different Rosetta protocols are used to model the TAPBPR G24-R36 loop: **(a)** cyclic coordinate descent (CCD), **(b)** kinematic closure (KIC), or **(c)** comparative modeling (CM). The template used was PDB ID 5WER where HLA-A*02:01 sequence was threaded onto H2-D^d using the partial_thread application in Rosetta. HLA-A*02:01 is green; TAPBPR is gray. The five lowest energy models of the TAPBPR G24-R36 loop from a total of 1,000 decoys calculated are shown in different colors. **(d)** C_α root-mean-square deviation (RMSD, Å) from the initial structure as a function of MD simulation time for the entire MHC-I/TAPBPR complex and for its individual components. **(e)** Model of the peptide-deficient HLA-A*02:01/hβ2m/TAPBPR complex at 0 nsec (start of the MD simulation) is shown in yellow. The dotted boxes highlight different loops present in TAPBPR. The range of conformations sampled by each TAPBPR loop captured at different times during the MD simulation are shown within each box. Only the TAPBPR loops are highlighted for visual clarity but the entire peptide-deficient HLA-A*02:01/hβ2m/TAPBPR complex was simulated.

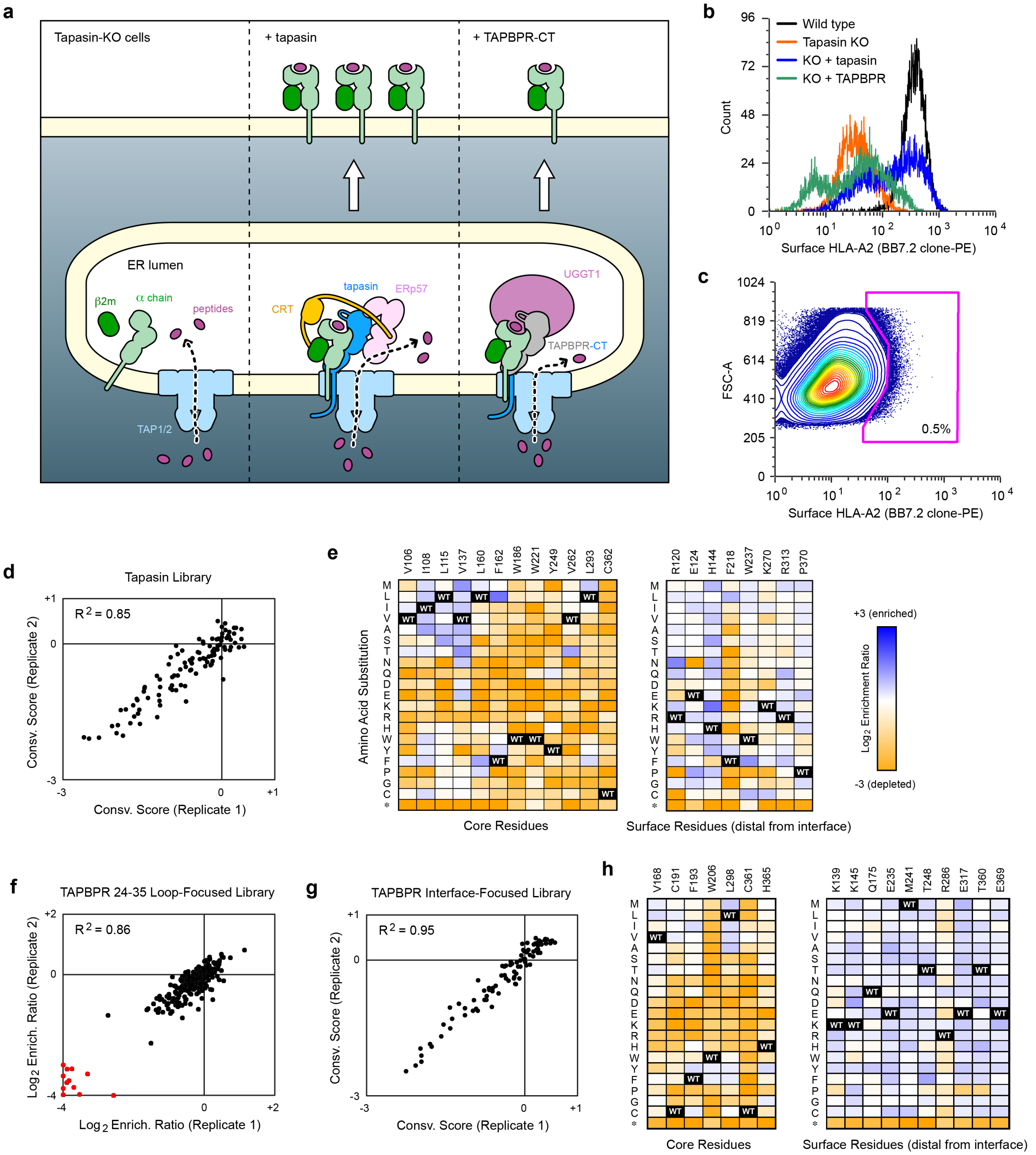

Supplementary Fig. 2. Selections based on functional replacement of endogenous tapasin for HLA-A2 processing and surface trafficking. (a) (*Left*) In tapasin-KO cells, HLA-A2 is poorly processed and loaded, and is retained intracellularly. (*Center*) Expression of tapasin from a plasmid rescues formation of the peptide-loading complex (PLC). HLA-A2 is now efficiently loaded with peptides, folds, and traffics to the plasma membrane. (*Right*) Expression of a chimera between the TAPBPR extracellular domains and the tapasin transmembrane and cytosolic domains partially rescues HLA-A2 processing. We hypothesize that the tapasin C-terminal tail might help bring TAPBPR and its associated glucosyltransferase UGGT1 in to a PLC-like complex with the TAP1/2

transporter. **(b)** Tapasin was knocked out by targeting indel mutations to exon 2 in Expi293F cells using Cas9/CRISPR-based editing (confirmed by deep sequencing genomic DNA; data deposited in GEO). Surface HLA-A*02:01 expression was substantially reduced in tapasin-KO cells (red) compared to wild type cells (black). Surface trafficking of HLA-A*02:01 was rescued by transfection with a plasmid encoding tapasin (blue), or partially rescued by over-expression of TAPBPR (green). When TAPBPR is over-expressed, a subset of cells also shows increased intracellular retention of HLA-A*02:01 (represented by the left-most peak in green). **(c)** Libraries of tapasin or TAPBPR-CT variants were expressed in tapasin-KO Expi293F cells, and after first gating by scattering and viability, the 0.5 % of cells with the highest levels of endogenous HLA-A2 at the cell surface were collected (magenta gate). Under these transfection conditions, cells typically express no more than a single sequence variant, and most cells are negative. The example shown here is following transfection of a TAPBPR library. **(d)** Conservation scores (calculated from the mean of the $\log_2$ enrichment ratios for each mutation at a residue position) following two independent selections of the tapasin library show close agreement. Tapasin residues with negative scores are tightly conserved for functional rescue of surface HLA-A*02:01. **(e)** Following selection of the tapasin library and deep sequencing, $\log_2$ enrichment ratios for mutations at control positions (in the core or at surface sites distal from the MHC-I interface) are plotted as heatmaps, colored from orange ($\leq$ -3, i.e. depleted/deleterious) to pale/white (0, i.e. neutral) to dark blue ($\geqslant$ +3, i.e. enriched). Due to read length limits for Illumina sequencing, enrichment of the wild type sequence in the experiment is unknown and shown in black. Mutated tapasin positions are on the horizontal axis, while amino acid substitutions are on the vertical axis (*, stop codon). **(f)** Following selection of a 24-35 loop-focused TAPBPR-CT library, $\log_2$ enrichment ratios for nonsynonymous mutations (black) in the 24-35 loop cluster near the origin. Nonsense mutations (red) are depleted. Data from two independent experiments show close agreement. **(g)** Conservation scores from the selection of a larger TAPBPR-CT library focused on the entire MHC-I interface are closely correlated between independent sorting experiments. **(h)** Heatmaps colored as in E, showing that core TAPBPR residues are generally restricted to hyrophobics (*left*) while surface TAPBPR residues distal from the MHC-I interface are mutationally tolerant (*right*).

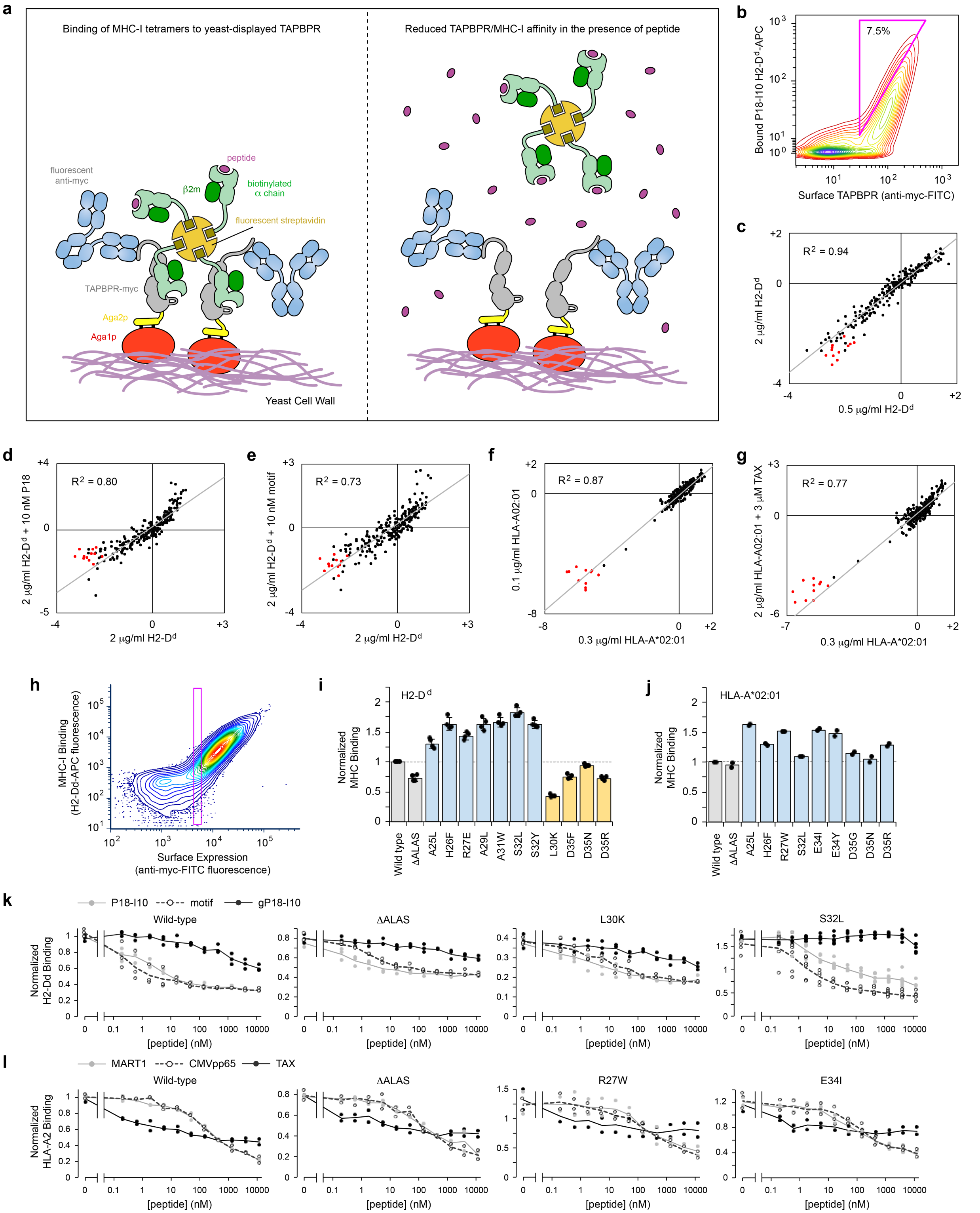

Supplementary Fig. 3. Yeast display for assessing how the sequence of the TAPBPR 24-35 loop influences binding to folded MHC-I. (a) The extracellular domains of TAPBPR are expressed on the surface of the yeast cell wall via an N-terminal fusion with Aga2p. A c-myc

epitope tag at the C-terminus is used for fluorescence detection of expression levels with anti-myc antibodies. MHC-I binding is measured by flow cytometry after incubation of the yeast with fluorescent pMHC-I tetramers. TAPBPR binds MHC-I with highest affinity when it is peptide free, and the addition of peptide reduces MHC-I binding to TAPBPR-expressing yeast. **(b)** The 24-35 loop of TAPBPR was diversified by saturation mutagenesis. The yeast-displayed library was sorted under a variety of conditions for binding to tetramers of P18-I10-loaded H2-D^d or TAX8-loaded HLA-A*02:01, in the presence or absence of free peptides P18-I10 or motif (for H2-D^d) or HTLV-1 TAX (for HLA-A*02:01). The gating strategy for one of the sorting experiments is shown. In this example, yeast were incubated with 0.5 μg/ml H2-D^d tetramer, and yeast expressing TAPBPR variants with the highest levels of binding were collected (magenta gate). MHC-I tetramer staining was below saturation (staining remained unsaturated up to at least 10 μg/ml tetramer). **(c-g)** Yeast-displayed TAPBPR libraries were sorted for binding to MHC-I tetramers and the enrichment or depletion of mutations were calculated following Illumina sequencing of the naive and sorted populations. Comparisons are shown between different sorting experiments for the enrichment ratios of nonsynonymous (black) and nonsense (red) mutations in the TAPBPR 24-35 loop. Data are highly correlated between replicate experiments using different concentrations of H2-D^d (c) or HLA-A*02:01 (f). Enrichment ratios are qualitatively similar when free peptides P18-I10 (d), motif (e), or TAX (g) are co-incubated with H2-D^d (d, e) or HLA-A*02:01 (g). **(h)** Yeast displaying surface TAPBPR were dual stained with P18-I10-loaded H2-D^d-APC tetramers and anti-myc-FITC for simultaneous detection by flow cytometry of bound MHC-I versus TAPBPR expression. Due to the use of avid MHC-I tetramers, binding to TAPBPR was found to be highly dependent on surface expression levels. To control for this, yeast were gated (magenta box) for low TAPBPR expression to minimize avidity effects and control for any differences in expression among TAPBPR mutants. Bound MHC-I (based on mean APC fluorescence) was measured within the magenta gate. **(i, j)** Validation by targeted mutagenesis of TAPBPR mutants predicted from the deep mutational scans to have reduced (orange) or increased (blue) binding to H2-D^d (I; 1.0 μg/ml, mean ± SD from n = 4 independent experiments) or HLA-A*02:01 (J; 2.0 μg/ml, mean ± range from n = 2 independent experiments). TAPBPR ΔALAS (grey) has slightly reduced MHC-I binding. Mean fluorescence for MHC-I binding is normalized to wild type TAPBPR (grey). **(k, l)** Binding of H2-D^d (K; 2.0 μg/ml, mean ± SD from n = 4 independent experiments) or HLA-A*02:01 (L; 2.0 μg/ml, mean ± range from n = 2 independent experiments) to TAPBPR-expressing yeast is competed by peptides. Shown are data from yeast expressing wild type TAPBPR, TAPBPR ΔALAS, and representative mutants. gP18-I10 is a low affinity peptide for H2-D^d missing an N-terminal anchoring residue for the A pocket. Peptide sequences are: RGPGRAFVTI (P18-I10), GPGRAFVTI (gP18-I10), AGPARAAAL (motif), LLFGYPVYV (HTLV-1 TAX9), ELAGIGILTV (MART1), NLVPMVATV (CMVpp65).

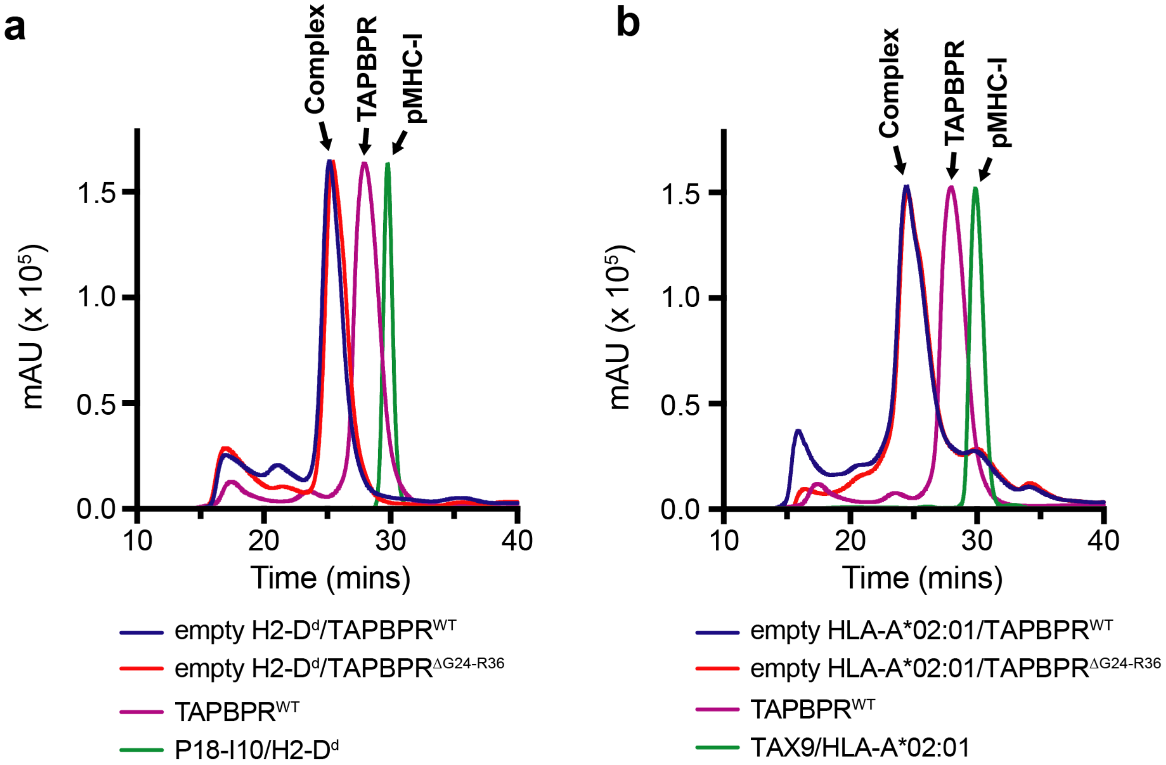

Supplementary Fig. 4. Purification of peptide-deficient MHC-I/TAPBPR complexes. Size exclusion chromatographs of peptide-deficient MHC-I/TAPBPR complexes, relative to the free pMHC-I and TAPBPR species. photoP18-I10/H2-D^{d}/hβ$_2$m **(a)** or photoFluM1/HLA-A*02:01/hβ$_2$m **(b)** were mixed with TAPBPR at 1:1 molar ratio and incubated for 1 hour at 25°C. The mixture was then UV-irradiated at 365 nm for 1 hour followed by centrifugation at 13,000 r.p.m. for 10 min to remove precipitates. Pure 1:1 stoichiometric H2-D^{d}/hβ$_2$m/TAPBPR **(a)** or HLA-A*02:01/hβ$_2$m/TAPBPR **(b)** complexes were isolated by SEC with a Superdex 200 Increase 10/300 GL column at flow rate of 0.5 mL/min in 50 mM NaCl, 20 mM sodium phosphate pH 7.2. The exact procedure was followed for complexes containing either TAPBPR^{WT} or TAPBPR$^{\Delta G24\text{-}R36}$, with chromatograms shown as different colors. Validation of peptide removal from the peaks at approx. 26 min corresponding to the MHC-I/TAPBPR complexes was achieved using liquid chromatography-mass spectroscopy (LC-MS) and carried out with passage through a Higgins PROTO300 C4 column (5 µm, 100 mm × 2.1 mm) followed by electron ion spray mass spectroscopy performed on a Thermo Finnigan LC-MS/MS (LTQ).

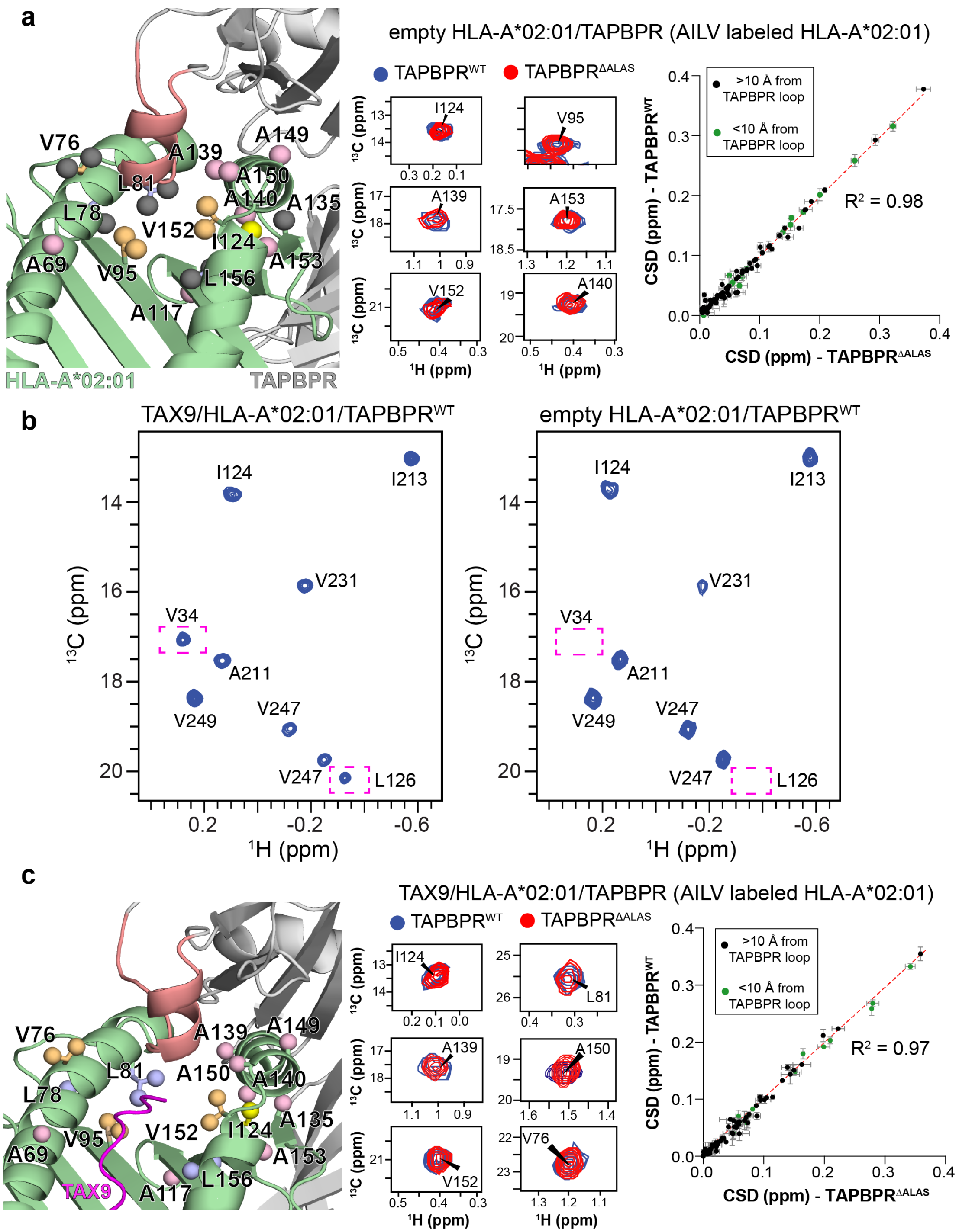

Supplementary Fig. 5. The TAPBPR G24-R36 loop does not form a stable interaction with the HLA-A*02:01 groove. (a) (Left) View of the peptide-deficient HLA-A*02:01/TAPBPR model (template PDB ID 5OPI) showing AILV methyl probes on HLA-A*02:01 (as spheres) within 10 Å angstroms of the TAPBPR G24-R36 loop (salmon). Methyl resonances of HLA-A*02:01 residues shown in black are missing in 2D ^{1}H-^{13}C methyl HMQC spectra of peptide-deficient HLA-A*02:01/hβ_2m/TAPBPR complex due to conformational exchange induced line broadening. (Middle) Zoom in of representative peaks from 2D ^{1}H-^{13}C methyl HMQC spectra of 80 µM peptide-deficient HLA-A*02:01 (AILV labeled)/hβ_2m in complex with TAPBPR^{WT} (red)

and TAPBPR$^{\Delta ALAS}$ (blue) recorded at 25°C at a ^{1}H field of 800 MHz. (Right) Comparison of chemical shift deviation (CSD) values measured between free TAX9/HLA-A*02:01/hβ_2m and peptide-deficient HLA-A*02:01/hβ_2m in complex with TAPBPR^{WT} or TAPBPR$^{\Delta ALAS}$. CSD values for HLA-A*02:01 methyl probes within 10 Å of the TAPBPR G24-R36 loop are shown in green, while those further than 10 Å are shown in black. The dotted red line is a linear fit of the data with R^2 value noted. **(b)** Comparison of 2D ^{1}H-^{13}C HMQC spectra of 80 μM TAX9/HLA-A*02:01 (AILV labeled)/hβ_2m/TAPBPR^{WT} in the presence of 200 μM excess TAPBPR^{WT} (left) with 80 μM peptide-deficient HLA-A*02:01 (AILV labeled)/hβ_2m/TAPBPR^{WT} (right). Dotted pink boxes highlight methyl resonances that become exchange broadened in the wild-type empty HLA-A*02:01/TAPBPR complex but are present in the wild-type TAX9/HLA-A*02:01/ complex. **(c)** (Left) View of the TAX9/HLA-A*02:01/TAPBPR model (template PDB ID 5OPI) showing AILV methyl probes on HLA-A*02:01 (as spheres) within 10 Å angstroms of the TAPBPR G24-R36 loop (salmon). (Middle) Zoom in of representative peaks from 2D ^{1}H-^{13}C methyl HMQC spectra of 80 μM TAX9/HLA-A*02:01 (AILV labeled)/hβ_2m in complex with TAPBPR^{WT} (red) and TAPBPR$^{\Delta ALAS}$ (blue) recorded at 25°C at a ^{1}H field of 800 MHz. (Right) Comparison of chemical shift deviation (CSD) values measured between free TAX9/HLA-A*02:01/hβ_2m and TAX9/HLA-A*02:01/hβ_2m in complex with TAPBPR^{WT} or TAPBPR$^{\Delta ALAS}$. CSD values for HLA-A*02:01 methyl probes within 10 Å of the TAPBPR G24-R36 loop are shown in green, while those further than 10 Å are shown in black. The dotted red line is a linear fit of the data with R^2 value noted. Scatter plot data points presented in panels **(a)** and **(c)** are the mean ± SD for n = 3 independent experimental replicates.

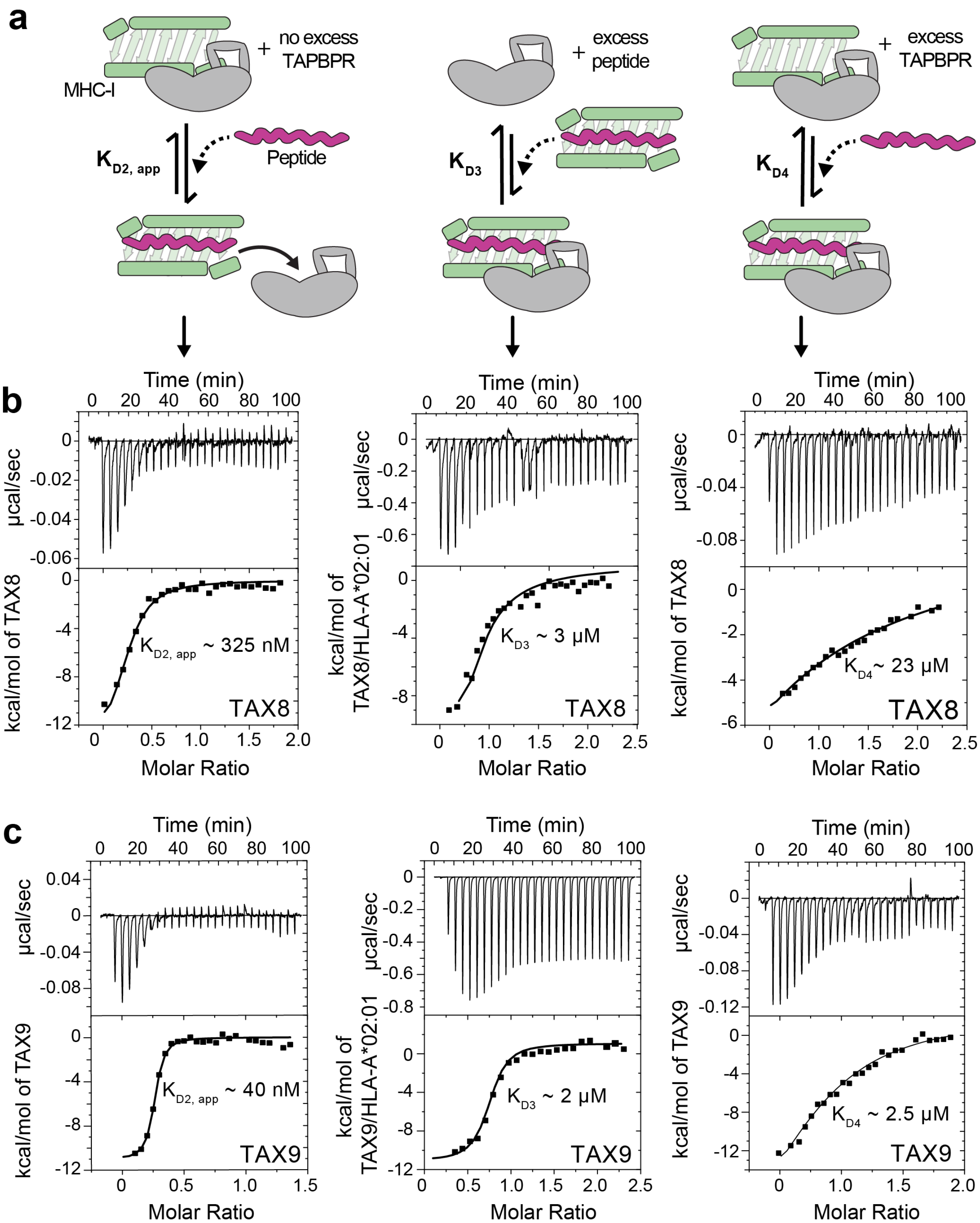

Supplementary Fig. 6. Design of ITC experiments to examine specific steps of the thermodynamic cycle of TAPBPR mediated peptide exchange. (a) Schematic of conditions in the calorimetry cell (top) and the syringe (titration represented by the dotted arrow) during ITC experiments for each step of the peptide exchange cycle. Due to experimental limitations, the measured K_{D2} is "apparent" and thus denoted with $K_{D2,\ app}$ **(b, c)** Representative examples of raw ITC data for each step of the peptide exchange cycle for TAX8 and TAX9 peptide. The line

represents the best fit of the data using a 1:1 model in Origin. Fitted apparent K_D values are reported. Details of the experiments are outline in the *Materials and Methods*.

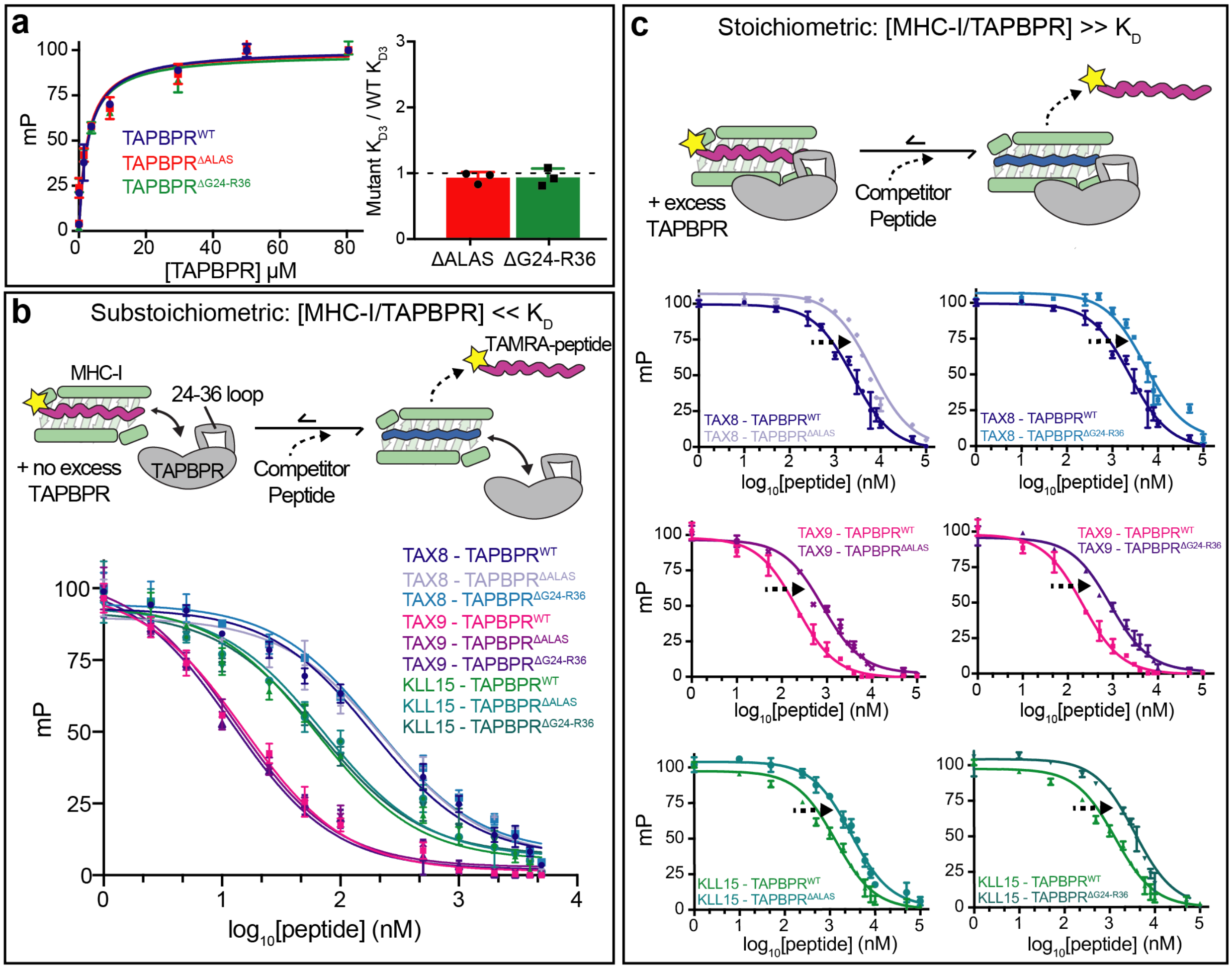

Supplementary Fig. 7. Design of FP assays to examine specific steps of the thermodynamic cycle of TAPBPR mediated peptide exchange. (a) FP experiments to probe the K_{D3} step of the peptide exchange cycle. (Left) Titration of graded concentrations of TAPBPR^{WT}, TAPBPR$^{\Delta ALAS}$ or TAPBPR$^{\Delta G24\text{-}R36}$ into 1 nM TAMRA-TAX9 and 50 nM TAX8/HLA-A*02:01/hβ$_2$m. (Right) Ratio of FP-determined K_{D3} values for mutant / wild-type TAPBPR. **(b)** Schematic of the design of FP experiments performed under substoichiometric conditions (no excess TAPBPR). Under substoichiometric conditions, 50 nM of wild-type or G24-R36 loop mutant peptide-deficient HLA-A*02:01/hβ$_2$m/TAPBPR complex is incubated with 1 nM TAMRA-TAX9 peptide and a range of concentrations of competitor peptide. millipolarization (mP) values are plotted as a function of the log$_{10}$ peptide concentration for TAX8, TAX9 and KLL15 peptides. **(c)** Schematic of the design of FP experiments performed under stoichiometric conditions (excess TAPBPR). Under stoichiometric conditions, 50 nM of wild-type or G24-R36 loop mutant peptide-deficient HLA-A*02:01/hβ$_2$m/TAPBPR complex is incubated with 1 nM TAMRA-TAX9 peptide, 2 μM TAPBPR (or TAPBPR$^{\Delta ALAS}$ or TAPBPR$^{\Delta G4\text{-}R36}$) and a range of concentrations of competitor peptide. mP values are plotted as a function of the log$_{10}$ peptide concentration for TAX8, TAX9 and KLL15 peptides. Data presented in panels **(a)**, **(b)** and **(c)** are mean ± SD for n = 3 independent experimental replicates.

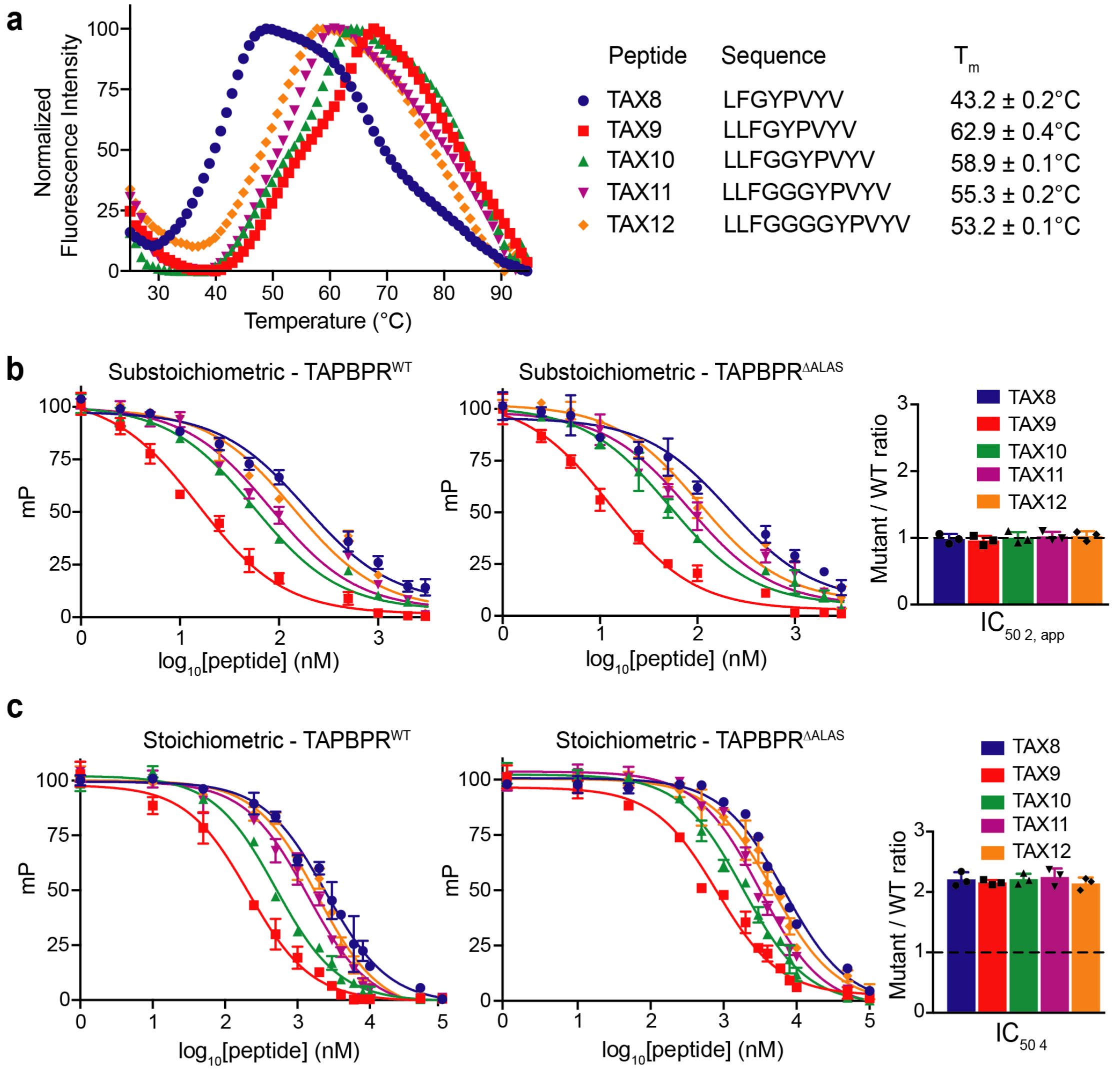

Supplementary Fig. 8. Evaluation of peptide length dependence for TAPBPR mediated exchange. (a) DSF experiments performed on 7 µM HLA-A*02:01/hβ_2m in complex with different TAX length variants. The peptide sequence and determined T_m values are shown. Fluorescence intensities were normalized for comparison. Data presented is the mean ± SD for n = 3 technical replicates. **(b)** (Left, Middle) FP performed under substoichiometric conditions, 50 nM of peptide-deficient HLA-A*02:01/hβ_2m/TAPBPR^{WT} or HLA-A*02:01/hβ_2m/TAPBPR$^{\Delta ALAS}$ complex is incubated with 1 nM TAMRA-TAX9 peptide and a range of concentrations of competitor peptide. millipolarization (mP) values are plotted as a function of the $\log_{10}$ peptide concentration for the TAX length variant peptides. (Right) Comparison of the ratio of FP determined $IC_{50\ 2,\ app}$ values for TAPBPR$^{\Delta ALAS}$ versus TAPBPR^{WT}. The dotted line represents no effect. (**c**) (Left, Middle) FP performed under stoichiometric conditions, 50 nM of peptide-deficient HLA-A*02:01/hβ_2m/TAPBPR^{WT} or HLA-A*02:01/hβ_2m/TAPBPR$^{\Delta ALAS}$ complex is incubated with 1 nM TAMRA-TAX9 peptide, 2 µM TAPBPR (or TAPBPR$^{\Delta ALAS}$) and a range of concentrations of competitor peptide. mP values are plotted as a function of the $\log_{10}$ peptide concentration for the TAX length variant peptides. (Right) Comparison of the ratio of FP determined $IC_{50\ 4}$ values for TAPBPR$^{\Delta ALAS}$ versus TAPBPR^{WT}. The dotted line represents no

effect. Data presented in panels **(b)** and **(c)** are mean ± SD for n = 3 independent experimental replicates.

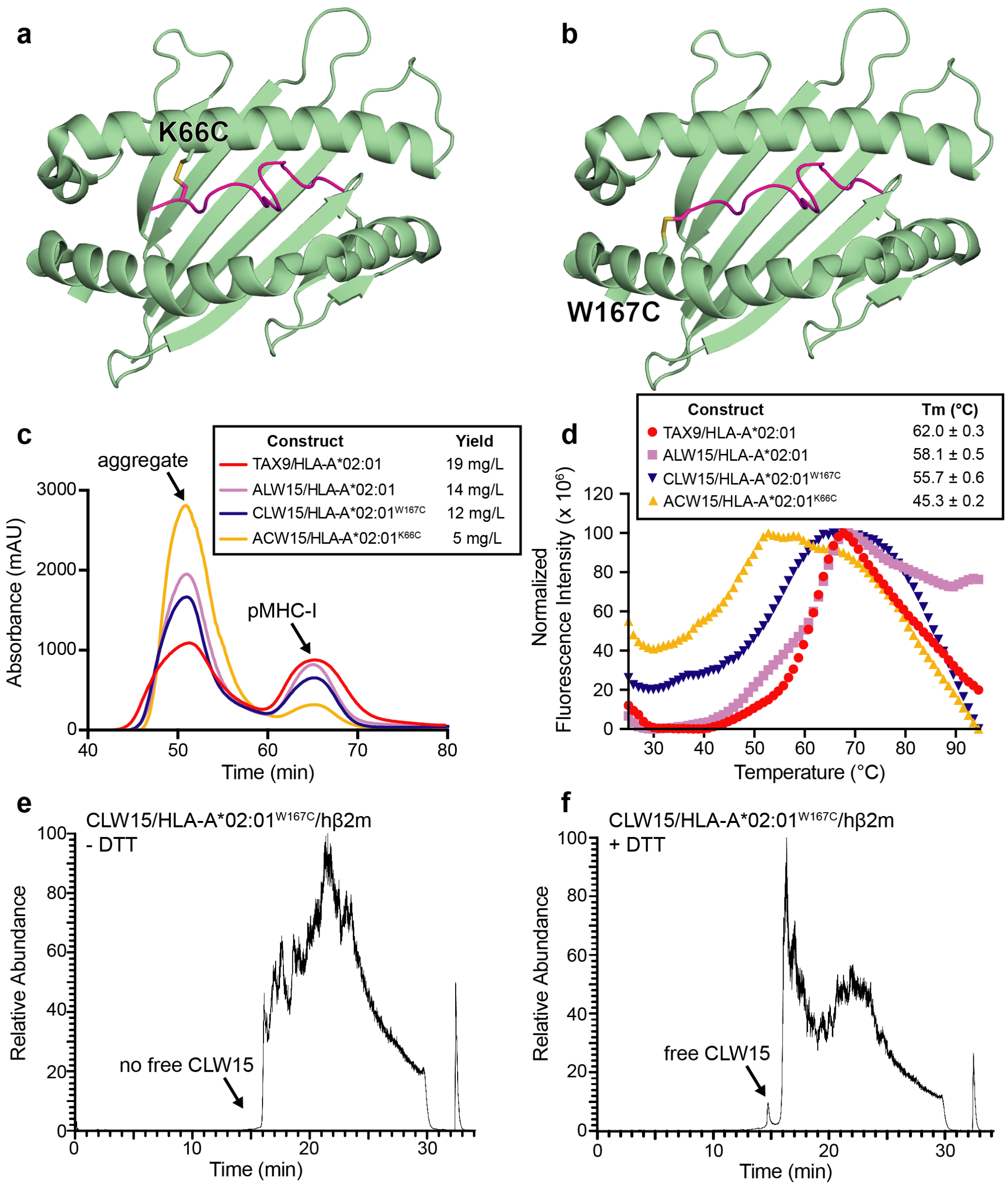

Supplementary Fig. 9. Design of disulfide mutants linking peptide to the MHC-I groove. Structural models of disulfides between ALW15 peptide (ALWDIETGQQKTVFV) and the HLA-A*02:01 groove based on Disulfide by Design v2. **(a)** Predicted disulfide between L2C of ALW15 (herein called ACW15) and K66C of HLA-A*02:01. **(b)** Predicted disulfide between A1C of ALW15 (herein called CLW15) and W167C of HLA-A*02:01. **(c)** Purification of *in vitro* refolded pMHC-I constructs by size exclusion chromatography. TAX9 is a high affinity peptide reference. ALW15 is the reference for the wild-type 15mer peptide. ACW15 and CLW15 peptides are the disulfide mutant designs shown in A. and B., respectively. The final refolded protein yield after purification is noted. **(d)** Differential scanning fluorimetry of purified pMHC-I constructs. The fitted melting temperature (Tm) is noted. Data presented is the mean ± SD for n = 3 technical replicates. **(e, f)** LC-MS of CLW15/HLA-A*02:01/hβ2m without and with 1 mM DTT.

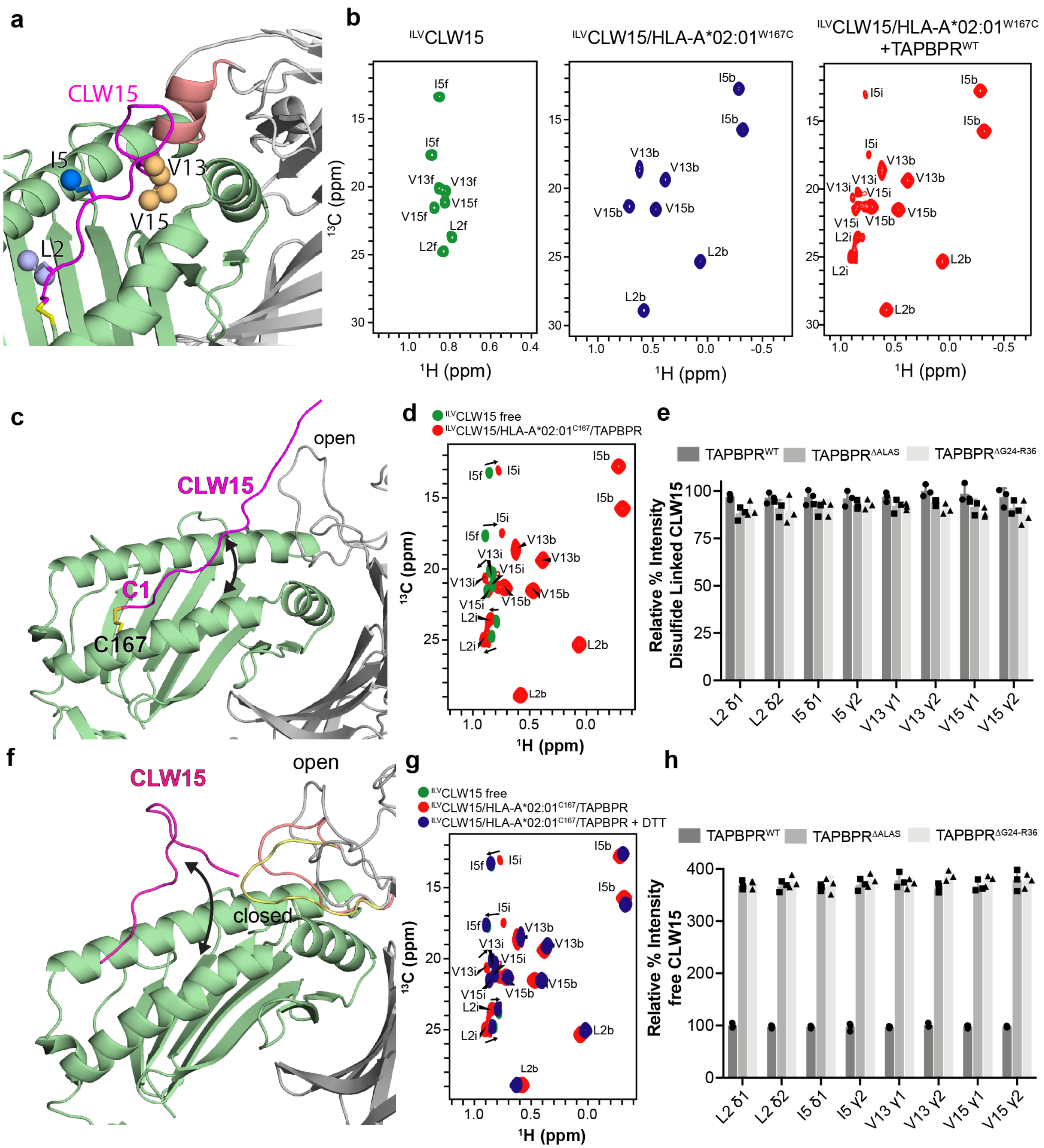

Supplementary Fig. 10. NMR characterization of chaperone mediated peptide exchange on MHC-I for a 15mer peptide. (a) Model of the CLW15/HLA-A*02:01/TAPBPR complex designed using Disulfide by Design v2 and RosettaCM. The disulfide is formed between C1 of CLW15 (CLWDIETGQQKTVFV) and W167C of HLA-A*02:01. The TAPBPR G24-R36 loop conformation (salmon) is from PDB ID 5OPI. ILV methyl residues of CLW15 are shown as spheres. CLW15 peptide is magenta, HLA-A*02:01 groove is green and TAPBPR is grey. **(b)** 2D ^{1}H-^{13}C methyl HMQC spectra of 100 μM ^{15}N/^{13}C ILV labeled CLW15 in the free state (green), in complex with HLA-A*02:01 (blue) and in complex with HLA-A*02:01 in the presence of 8-fold excess TAPBPR (red) recorded at 25 °C at a ^{1}H field of 800 MHz. Methyl resonances in the pMHC-I state are denoted "b" for bound. Methyl resonances in the pMHC-I/TAPBPR state are

denoted "i" for intermediate. Methyl resonances for free CLW15 are denoted "f" for free. **(c)** Model of the CLW15/HLA-A*02:01/TAPBPR complex where CLW15 samples an extended conformation and the TAPBPR G24-R36 loop samples an open conformation. The modeled protein conformations were obtained from MD simulations. **(d)** Overlay of 2D ^{1}H-^{13}C methyl HMQC spectra of 100 μM ^{15}N/^{13}C ILV labeled CLW15 in the free state (green) and in complex with HLA-A*02:01 in the presence of 8-fold excess TAPBPR (red) recorded at 25 °C at a ^{1}H field of 800 MHz. Methyl resonances in the pMHC-I state are denoted as in panel **(b)**. **(e)** Comparison of NMR signal intensity for each methyl resonance of disulfide linked CLW15 intermediate in the pMHC-I/TAPBPR state with and without the TAPBPR G24-R36 loop. Data presented are mean ± SD for n = 3 independent experimental replicates. **(f)** Model of the CLW15/HLA-A*02:01/TAPBPR complex is shown when the disulfide between CLW15 and HLA-A*02:01 is reduced with DTT. The open and closed conformations of the TAPBPR G24-R36 loop obtained from MD simulations are shown in grey, salmon and yellow. **(g)** 2D ^{1}H-^{13}C methyl HMQC spectra of 100 μM ^{15}N/^{13}C ILV labeled CLW15 in the free state (green), in complex with HLA-A*02:01 in the presence of 8-fold excess TAPBPR (red) and in complex with HLA-A*02:01 in the presence of 8-fold excess TAPBPR and 1 mM DTT (blue) recorded at 25 °C at a ^{1}H field of 800 MHz. Methyl resonances in the pMHC-I state are denoted as in panel **(b). (h)** Comparison of NMR signal intensity for each methyl resonance of free CLW15 peptide when released from the MHC-I/TAPBPR complex with and without the TAPBPR G24-R36 loop. Data presented are mean ± SD for n = 3 independent experimental replicates.

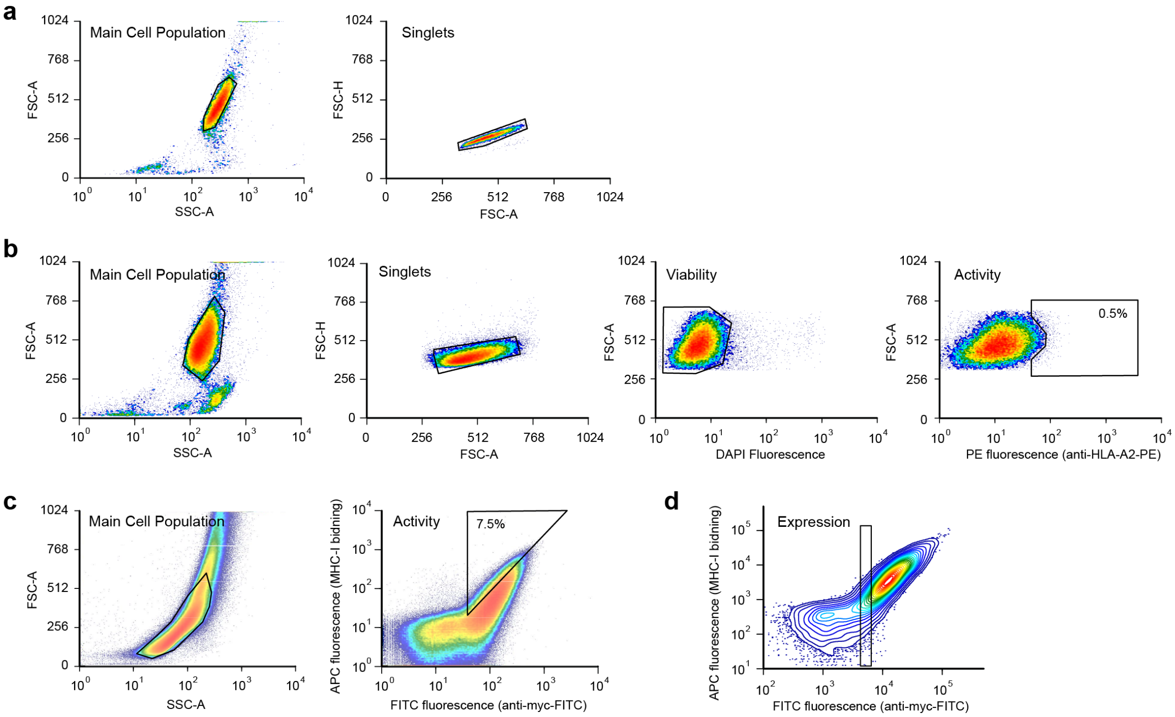

Supplementary Fig. 11. Gating strategies for flow cytometry experiments. In all cases, a series of sequential gates, shown as black polygons in the plots from left to right, were drawn to include events meeting the criteria. **(a)** For analysis of transfected Expi239F cells described in Fig. 2a, 2d, and 2h, cells were first gated by FSC-A/SSC-A for the main population. Cells were then gated by FSC-A/FSC-H to remove doublets. **(b)** For FACS selections of tapasin or TAPBPR transfected Expi293F cells (used for the deep mutational scans presented in Fig. 2b-c, 2e-g, and Supplementary Fig. 2), cells were gated for the main population based on FSC-A/SSC-A, followed by gating on FSC-A/FSC-H to exclude doublets. DAPI-negative events were gated to exclude DAPI-positive dead cells. The cells with the highest surface HLA-A2 expression (top 0.5%) were then collected by gating on FSC-A versus PE fluorescence. **(c)** For deep mutagenesis of TAPBPR using yeast surface display (mutational data shown in Fig. 3 and Supplementary Fig. 3b-g), yeast were gated for the main population by FSC-A/SSC-A, followed by gating on the top 7.5% of cells for APC fluorescence (i.e. binding of fluorescently labeled MHC-I tetramers) with respect to FITC fluorescence (i.e. TAPBPR surface display based on epitope tag detection with anti-myc-FITC). **(d)** For analysis of MHC-I tetramer binding to yeast displaying TAPBPR (Supplementary Fig. 3h-l), events were first gated by FSC-A/SSC-A (left plot in panel c) and then gated by FITC fluorescence for a low level of TAPBPR expression that was consistent across all samples.

**Supplementary Table 1. Nucleotide sequence of codon-optimized human tapasin with an extracellular FLAG epitope tag (insert in Addgene Plasmid # 141308)**
ATGGCCGTCATGGCGCCCCGAACCCTCGTCCTGCTACTCTCGGGGGCCCTGGCCCT<br>GACCCAGACCTGGGCGGGCTCCGACTACAAGGACGATGATGACAAGGGAGGATC<br>CGGCCCTGCCGTAATCGAGTGTTGGTTTGTTGAAGACGCTAGTGGTAAAGGGCTG<br>GCTAAACGGCCAGGCGCACTTCTGTTGCGGCAAGGTCCGGGAGAACCACCTCCAC<br>GACCCGACTTGGACCCTGAATTGTACCTCTCTGTACACGACCCAGCTGGAGCACTC<br>CAAGCTGCTTTCCGACGGTACCCAAGAGGTGCTCCAGCACCGCATTGCGAGATGA<br>GTCGGTTCGTTCCTTTGCCTGCGAGTGCAAAGTGGGCATCAGGTCTGACACCAGCA<br>CAAAACTGTCCACGAGCGCTTGATGGAGCGTGGCTGATGGTTTCCATAAGCTCTCC<br>GGTGCTTTCACTGTCATCACTGCTCAGGCCACAACCTGAACCCCAACAAGAACCC<br>GTACTGATAACAATGGCGACGGTAGTCCTTACGGTCCTTACGCATACACCAGCAC<br>CCAGGGTGCGATTGGGACAAGACGCACTCCTCGACCTTAGCTTTGCGTACATGCC<br>ACCAACCTCAGAGGCGGCTTCCTCTCTTGCACCAGGACCACCACCGTTCGGCCTTG<br>AATGGAGACGACAGCACTTGGGGAAGGGCCATTTGCTGTTGGCTGCTACCCCAGG<br>GCTGAACGGGCAGATGCCGGCAGCACAAGAGGGAGCCGTAGCTTTTGCTGCGTGG<br>GATGATGACGAACCATGGGGACCATGGACTGGAAACGGGACTTTCTGGCTGCCAA<br>CAGTACAGCCCTTTCAAGAAGGAACGTATCTGGCCACCATCCATTTGCCTTATCTG<br>CAAGGCCAGGTCACACTTGAACTCGCTGTCTATAAACCGCCTAAGGTGAGTCTCA<br>TGCCCGCAACTTTGGCTAGGGCAGCACCTGGCGAAGCTCCACCTGAATTGCTCTGT<br>TTGGTATCCCATTTCTACCCCAGTGGTGGCCTTGAAGTGGAATGGGAACTTAGAGG<br>AGGACCAGGTGGTCGATCTCAGAAAGCTGAAGGGCAGCGATGGCTCTCCGCTCTC<br>AGGCACCACTCAGACGGATCTGTATCACTGTCAGGGCATTTGCAGCCACCGCCAG<br>TTACCACCGAGCAGCATGGAGCTCGGTATGCGTGTCGCATTCACCACCCTAGCCTT<br>CCGGCGTCAGGAAGATCAGCGGAGGTAACACTTGAGGTCGCTGGGTTGTCCGGAC<br>CCAGCTTGGAGGACAGTGTTGGTCTGTTCCTGTCCGCTTTCCTGTTGCTGGGTCTGT<br>TTAAGGCACTCGGATGGGCAGCAGTTTATCTCAGCACCTGTAAAGACAGCAAGAA<br>AAAGGCAGAATAG

Supplementary Table 2. Sequences of primers used in this study.	
Primer Name	Sequence
TAPBPR_101	TGC TGA CTG CAG TGG GAA GNN KGT GAC CTG TGA GAT CTC CCG
TAPBPR_103	TGC AGT GGG AAG GAG GTG NNK TGT GAG ATC TCC CGC TAC T
TAPBPR_105	TGG GAA GGA GGT GAC CTG TNN KAT CTC CCG CTA CTT TCT CCA
TAPBPR_107	AAG GAG GTG ACC TGT GAG ATC NNK CGC TAC TTT CTC CAG ATG ACA
TAPBPR_108	GAG GTG ACC TGT GAG ATC TCC NNK TAC TTT CTC CAG ATG ACA GAG AC
TAPBPR_109	GAC CTG TGA GAT CTC CCG CNN KTT TCT CCA GAT GAC AGA GAC CA
TAPBPR_110	CCT GTG AGA TCT CCC GCT ACN NKC TCC AGA TGA CAG AGA CCA C
TAPBPR_111	CTG TGA GAT CTC CCG CTA CTT TNN KCA GAT GAC AGA GAC CAC TGT T
TAPBPR_112	TGA GAT CTC CCG CTA CTT TCT CNN KAT GAC AGA GAC CAC TGT TAA GAC
TAPBPR_115	CCG CTA CTT TCT CCA GAT GAC ANN KAC CAC TGT TAA GAC AGC AGC
TAPBPR_125	TGT TAA GAC AGC AGC TTG GTT CNN KGC CAA CGT GCA GGT CT
TAPBPR_126	AGA CAG CAG CTT GGT TCA TGN NKA ACG TGC AGG TCT CTG GA
TAPBPR_127	AGC AGC TTG GTT CAT GGC CNN KGT GCA GGT CTC TGG AGG G
TAPBPR_128	AGC TTG GTT CAT GGC CAA CNN KCA GGT CTC TGG AGG GGG
TAPBPR_129	TTG GTT CAT GGC CAA CGT GNN KGT CTC TGG AGG GGG ACC
TAPBPR_130	TTC ATG GCC AAC GTG CAG NNK TCT GGA GGG GGA CCT AGC
TAPBPR_131	ATG GCC AAC GTG CAG GTC NNK GGA GGG GGA CCT AGC ATC
TAPBPR_136	GTC TCT GGA GGG GGA CCT NNK ATC TCC TTG GTG ATG AAG ACT C
TAPBPR_137	TCT GGA GGG GGA CCT AGC NNK TCC TTG GTG ATG AAG ACT CCC
TAPBPR_138	TGG AGG GGG ACC TAG CAT CNN KTT GGT GAT GAA GAC TCC CAG G
TAPBPR_180	TCC AGG TGA TGA CAC AGA CCN NKT CCC TGA GCT TCC TGC T
TAPBPR_181	AGG TGA TGA CAC AGA CCC AAN NKC TGA GCT TCC TGC TGG G
TAPBPR_186	CCC AAT CCC TGA GCT TCC TGN NKG GGT CCT CAG CCT CCT T
TAPBPR_210	ACC TCA TCA GTG TGG AGT GGN NKC TGC AGC ACA AGG GCA
TAPBPR_212	AGT GTG GAG TGG CGA CTG NNK CAC AAG GGC AGG GGT CA
TAPBPR_213	GTG GAG TGG CGA CTG CAG NNK AAG GGC AGG GGT CAG TT
TAPBPR_214	GAG TGG CGA CTG CAG CAC NNK GGC AGG GGT CAG TTG GT
TAPBPR_215	TGG CGA CTG CAG CAC AAG NNK AGG GGT CAG TTG GTG TAC A
TAPBPR_216	CGA CTG CAG CAC AAG GGC NNK GGT CAG TTG GTG TAC AGC T
TAPBPR_217	CTG CAG CAC AAG GGC AGG NNK CAG TTG GTG TAC AGC TGG A
TAPBPR_257	ACC CTG CCC GGC CTC ACT NNK CAG GAC GAG GGG ACC TAC
TAPBPR_262	CTC ACT ATA CAG GAC GAG GGG NNK TAC ATT TGC CAG ATC ACC ACC
TAPBPR_263	TAT ACA GGA CGA GGG GAC CNN KAT TTG CCA GAT CAC CAC CTC
TAPBPR_264	ACA GGA CGA GGG GAC CTA CNN KTG CCA GAT CAC CAC CTC T
TAPBPR_266	ACG AGG GGA CCT ACA TTT GCN NKA TCA CCA CCT CTC TGT ACC G
TAPBPR_273	CCA GAT CAC CAC CTC TCT GTA CNN KGC TCA GCA GAT CAT CCA GC
TAPBPR_275	CCA CCT CTC TGT ACC GAG CTN NKC AGA TCA TCC AGC TCA ACA TCC
TAPBPR_276	ACC TCT CTG TAC CGA GCT CAG NNK ATC ATC CAG CTC AAC ATC CAA
TAPBPR_277	TCT GTA CCG AGC TCA GCA GNN KAT CCA GCT CAA CAT CCA AGC
TAPBPR_279	ACC GAG CTC AGC AGA TCA TCN NKC TCA ACA TCC AAG CTT CCC C
TAPBPR_281	GCT CAG CAG ATC ATC CAG CTC NNK ATC CAA GCT TCC CCT AAA GTA C
TAPBPR_284	GAT CAT CCA GCT CAA CAT CCA ANN KTC CCC TAA AGT ACG ACT GAG C
TAPBPR_306	CCA CCC TCA TCT GCG ACA TTN NKG GCT ATT ACC CTC TGG ATG TG
TAPBPR_309	CTG CGA CAT TGC TGG CTA TNN KCC TCT GGA TGT GGT GGT G
TAPBPR_310	TGC GAC ATT GCT GGC TAT TAC NNK CTG GAT GTG GTG GTG ACG
TAPBPR_311	CGA CAT TGC TGG CTA TTA CCC TNN KGA TGT GGT GGT GAC GTG G
TAPBPR_312	ATT GCT GGC TAT TAC CCT CTG NNK GTG GTG GTG ACG TGG AC
TAPBPR_313	TGC TGG CTA TTA CCC TCT GGA TNN KGT GGT GAC GTG GAC CC
TAPBPR_314	TGG CTA TTA CCC TCT GGA TGT GNN KGT GAC GTG GAC CCG AGA
TAPBPR_315	GCT ATT ACC CTC TGG ATG TGG TGN NKA CGT GGA CCC GAG AGG
TAPBPR_316	CCC TCT GGA TGT GGT GGT GNN KTG GAC CCG AGA GGA GC
TAPBPR_328	CTG GGT GGA TCC CCA GCC NNK GTC TCT GGT GCC TCC TTC T
TAPBPR_329	GGT GGA TCC CCA GCC CAA NNK TCT GGT GCC TCC TTC TCC
TAPBPR_330	TGG ATC CCC AGC CCA AGT CNN KGG TGC CTC CTT CTC CAG C
TAPBPR_331	TCC CCA GCC CAA GTC TCT NNK GCC TCC TTC TCC AGC CT
TAPBPR_332	CCC AGC CCA AGT CTC TGG TNN KTC CTT CTC CAG CCT CAG G
TAPBPR_333	GCC CAA GTC TCT GGT GCC NNK TTC TCC AGC CTC AGG CA
TAPBPR_334	CCA AGT CTC TGG TGC CTC CNN KTC CAG CCT CAG GCA AAG C
TAPBPR_335	AAG TCT CTG GTG CCT CCT TCN NKA GCC TCA GGC AAA GCG
TAPBPR_336	CTC TGG TGC CTC CTT CTC CNN KCT CAG GCA AAG CGT GGC
TAPBPR_337	GGT GCC TCC TTC TCC AGC NNK AGG CAA AGC GTG GCA G
TAPBPR_338	TGC CTC CTT CTC CAG CCT CNN KCA AAG CGT GGC AGG CA
TAPBPR_339	CTC CTT CTC CAG CCT CAG GNN KAG CGT GGC AGG CAC C
TAPBPR_340	TTC TCC AGC CTC AGG CAA NNK GTG GCA GGC ACC TAC AG

TAPBPR_341	TCC AGC CTC AGG CAA AGC NNK GCA GGC ACC TAC AGC AT
TAPBPR_342	AGC CTC AGG CAA AGC GTG NNK GGC ACC TAC AGC ATC TCC
TAPBPR_343	CTC AGG CAA AGC GTG GCA NNK ACC TAC AGC ATC TCC TCC TC
TAPBPR_344	AGG CAA AGC GTG GCA GGC NNK TAC AGC ATC TCC TCC TCT CTC
TAPBPR_345	CAA AGC GTG GCA GGC ACC NNK AGC ATC TCC TCC TCT CTC AC
TAPBPR_346	AGC GTG GCA GGC ACC TAC NNK ATC TCC TCC TCT CTC ACC G
TAPBPR_347	GTG GCA GGC ACC TAC AGC NNK TCC TCC TCT CTC ACC GC
TAPBPR_348	GGC AGG CAC CTA CAG CAT CNN KTC CTC TCT CAC CGC AGA A
TAPBPR_350	GGC ACC TAC AGC ATC TCC TCC NNK CTC ACC GCA GAA CCT GG
TAPBPR_352	TAC AGC ATC TCC TCC TCT CTC NNK GCA GAA CCT GGC TCT GC
TAPBPR_369	ACC TGC CAG GTC ACA CAC NNK TCT CTG GAG GAG CCC CT
TAPBPR_142	ACC TAG CAT CTC CTT GGT GAT GNN KAC TCC CAG GGT CGC C
TAPBPR_148	AAG ACT CCC AGG GTC GCC NNK AAT GAG GTG CTC TGG CAC
TAPBPR_178	GTG GAG TTC CAG GTG ATG ACA NNK ACC CAA TCC CTG AGC TTC C
TAPBPR_238	CGG AAG GGC GCT ACC CTG NNK CCT GCA CAA CTG GGC A
TAPBPR_244	GAG CCT GCA CAA CTG GGC NNK GCC AGG GAT GCC TCC C
TAPBPR_251	GCC AGG GAT GCC TCC CTC NNK CTG CCC GGC CTC ACT
TAPBPR_289	ACA TCC AAG CTT CCC CTA AAG TAN NKC TGA GCT TGG CAA ACG AAG
TAPBPR_320	GTG GTG ACG TGG ACC CGA NNK GAG CTG GGT GGA TCC CC
TAPBPR_363	CTC TGC AGG TGC CAC TTA CNN KTG CCA GGT CAC ACA CAT C
TAPBPR_372	CCA GGT CAC ACA CAT CTC TCT GNN KGA GCC CCT TGG GGC C
TAPBPR_171	GGG ACT GTG CGA ACT GCA NNK GAG TTC CAG GTG ATG ACA CA
TAPBPR_194	GTC CTC AGC CTC CTT GGA CNN KGG CTT CTC CAT GGC ACC
TAPBPR_196	GCC TCC TTG GAC TGT GGC NNK TCC ATG GCA CCG GGC
TAPBPR_209	TGG ACC TCA TCA GTG TGG AGN NKC GAC TGC AGC ACA AGG G
TAPBPR_301	GAA GCT CTG CTG CCC ACC NNK ATC TGC GAC ATT GCT GGC
TAPBPR_364	TGC AGG TGC CAC TTA CAC CNN KCA GGT CAC ACA CAT CTC TCT G
TAPBPR_368	TAC ACC TGC CAG GTC ACA NNK ATC TCT CTG GAG GAG CCC
TAPBPR_101R	CTT CCC ACT GCA GTC AGC A
TAPBPR_103R	CAC CTC CTT CCC ACT GCA
TAPBPR_105R	ACA GGT CAC CTC CTT CCC A
TAPBPR_107R	GAT CTC ACA GGT CAC CTC CTT
TAPBPR_108R	GGA GAT CTC ACA GGT CAC CTC
TAPBPR_109R	GCG GGA GAT CTC ACA GGT C
TAPBPR_110R	GTA GCG GGA GAT CTC ACA GG
TAPBPR_111R	AAA GTA GCG GGA GAT CTC ACA G
TAPBPR_112R	GAG AAA GTA GCG GGA GAT CTC A
TAPBPR_115R	TGT CAT CTG GAG AAA GTA GCG G
TAPBPR_125R	GAA CCA AGC TGC TGT CTT AAC A
TAPBPR_126R	CAT GAA CCA AGC TGC TGT CT
TAPBPR_127R	GGC CAT GAA CCA AGC TGC T
TAPBPR_128R	GTT GGC CAT GAA CCA AGC T
TAPBPR_129R	CAC GTT GGC CAT GAA CCA A
TAPBPR_130R	CTG CAC GTT GGC CAT GAA
TAPBPR_131R	GAC CTG CAC GTT GGC CAT
TAPBPR_136R	AGG TCC CCC TCC AGA GAC
TAPBPR_137R	GCT AGG TCC CCC TCC AGA
TAPBPR_138R	GAT GCT AGG TCC CCC TCC A
TAPBPR_180R	GGT CTG TGT CAT CAC CTG GA
TAPBPR_181R	TTG GGT CTG TGT CAT CAC CT
TAPBPR_186R	CAG GAA GCT CAG GGA TTG GG
TAPBPR_210R	CCA CTC CAC ACT GAT GAG GT
TAPBPR_212R	CAG TCG CCA CTC CAC ACT
TAPBPR_213R	CTG CAG TCG CCA CTC CAC
TAPBPR_214R	GTG CTG CAG TCG CCA CTC
TAPBPR_215R	CTT GTG CTG CAG TCG CCA
TAPBPR_216R	GCC CTT GTG CTG CAG TCG
TAPBPR_217R	CCT GCC CTT GTG CTG CAG
TAPBPR_257R	AGT GAG GCC GGG CAG GGT
TAPBPR_262R	CCC CTC GTC CTG TAT AGT GAG
TAPBPR_263R	GGT CCC CTC GTC CTG TAT A
TAPBPR_264R	GTA GGT CCC CTC GTC CTG T
TAPBPR_266R	GCA AAT GTA GGT CCC CTC GT
TAPBPR_273R	GTA CAG AGA GGT GGT GAT CTG G
TAPBPR_275R	AGC TCG GTA CAG AGA GGT GG
TAPBPR_276R	CTG AGC TCG GTA CAG AGA GGT

TAPBPR_277R	CTG CTG AGC TCG GTA CAG A
TAPBPR_279R	GAT GAT CTG CTG AGC TCG GT
TAPBPR_281R	GAG CTG GAT GAT CTG CTG AGC
TAPBPR_284R	TTG GAT GTT GAG CTG GAT GAT C
TAPBPR_306R	AAT GTC GCA GAT GAG GGT GG
TAPBPR_309R	ATA GCC AGC AAT GTC GCA G
TAPBPR_310R	GTA ATA GCC AGC AAT GTC GCA
TAPBPR_311R	AGG GTA ATA GCC AGC AAT GTC G
TAPBPR_312R	CAG AGG GTA ATA GCC AGC AAT
TAPBPR_313R	ATC CAG AGG GTA ATA GCC AGC A
TAPBPR_314R	CAC ATC CAG AGG GTA ATA GCC A
TAPBPR_315R	CAC CAC ATC CAG AGG GTA ATA GC
TAPBPR_316R	CAC CAC CAC ATC CAG AGG G
TAPBPR_328R	GGC TGG GGA TCC ACC CAG
TAPBPR_329R	TTG GGC TGG GGA TCC ACC
TAPBPR_330R	GAC TTG GGC TGG GGA TCC A
TAPBPR_331R	AGA GAC TTG GGC TGG GGA
TAPBPR_332R	ACC AGA GAC TTG GGC TGG G
TAPBPR_333R	GGC ACC AGA GAC TTG GGC
TAPBPR_334R	GGA GGC ACC AGA GAC TTG G
TAPBPR_335R	GAA GGA GGC ACC AGA GAC TT
TAPBPR_336R	GGA GAA GGA GGC ACC AGA G
TAPBPR_337R	GCT GGA GAA GGA GGC ACC
TAPBPR_338R	GAG GCT GGA GAA GGA GGC A
TAPBPR_339R	CCT GAG GCT GGA GAA GGA G
TAPBPR_340R	TTG CCT GAG GCT GGA GAA
TAPBPR_341R	GCT TTG CCT GAG GCT GGA
TAPBPR_342R	CAC GCT TTG CCT GAG GCT
TAPBPR_343R	TGC CAC GCT TTG CCT GAG
TAPBPR_344R	GCC TGC CAC GCT TTG CCT
TAPBPR_345R	GGT GCC TGC CAC GCT TTG
TAPBPR_346R	GTA GGT GCC TGC CAC GCT
TAPBPR_347R	GCT GTA GGT GCC TGC CAC
TAPBPR_348R	GAT GCT GTA GGT GCC TGC C
TAPBPR_350R	GGA GGA GAT GCT GTA GGT GCC
TAPBPR_352R	GAG AGA GGA GGA GAT GCT GTA
TAPBPR_369R	GTG TGT GAC CTG GCA GGT
TAPBPR_142R	CAT CAC CAA GGA GAT GCT AGG T
TAPBPR_148R	GGC GAC CCT GGG AGT CTT
TAPBPR_178R	TGT CAT CAC CTG GAA CTC CAC
TAPBPR_238R	CAG GGT AGC GCC CTT CCG
TAPBPR_244R	GCC CAG TTG TGC AGG CTC
TAPBPR_251R	GAG GGA GGC ATC CCT GGC
TAPBPR_289R	TAC TTT AGG GGA AGC TTG GAT GT
TAPBPR_320R	TCG GGT CCA CGT CAC CAC
TAPBPR_363R	GTA AGT GGC ACC TGC AGA G
TAPBPR_372R	CAG AGA GAT GTG TGT GAC CTG G
TAPBPR_171R	TGC AGT TCG CAC AGT CCC
TAPBPR_194R	GTC CAA GGA GGC TGA GGA C
TAPBPR_196R	GCC ACA GTC CAA GGA GGC
TAPBPR_209R	CTC CAC ACT GAT GAG GTC CA
TAPBPR_301R	GGT GGG CAG CAG AGC TTC
TAPBPR_364R	GGT GTA AGT GGC ACC TGC A
TAPBPR_368R	TGT GAC CTG GCA GGT GTA
tapasin_iso1_33	TCG AGT GTT GGT TTG TTG AAG ACN NKA GTG GTA AAG GGC TGG CT
tapasin_iso1_34	TGT TGG TTT GTT GAA GAC GCT NNK GGT AAA GGG CTG GCT AAA CG
tapasin_iso1_35	TTG GTT TGT TGA AGA CGC TAG TNN KAA AGG GCT GGC TAA ACG G
tapasin_iso1_36	TTT GTT GAA GAC GCT AGT GGT NNK GGG CTG GCT AAA CGG C
tapasin_iso1_37	TTG TTG AAG ACG CTA GTG GTA AAN NKC TGG CTA AAC GGC CAG G
tapasin_iso1_38	GAA GAC GCT AGT GGT AAA GGG NNK GCT AAA CGG CCA GGC G
tapasin_iso1_39	ACG CTA GTG GTA AAG GGC TGN NKA AAC GGC CAG GCG C
tapasin_iso1_88	TAC CCA AGA GGT GCT CCA NNK CCG CAT TGC GAG ATG AGT
tapasin_iso1_90	AGA GGT GCT CCA GCA CCG NNK TGC GAG ATG AGT CGG TTC
tapasin_iso1_92	GCT CCA GCA CCG CAT TGC NNK ATG AGT CGG TTC GTT CCT TT
tapasin_iso1_94	GCA CCG CAT TGC GAG ATG NNK CGG TTC GTT CCT TTG CCT
tapasin_iso1_95	ACC GCA TTG CGA GAT GAG TNN KTT CGT TCC TTT GCC TGC G

tapasin_iso1_96	CGC ATT GCG AGA TGA GTC GGN NKG TTC CTT TGC CTG CGA GT
tapasin_iso1_97	TTG CGA GAT GAG TCG GTT CNN KCC TTT GCC TGC GAG TGC
tapasin_iso1_98	GCG AGA TGA GTC GGT TCG TTN NKT TGC CTG CGA GTG CAA
tapasin_iso1_99	AGA TGA GTC GGT TCG TTC CTN NKC CTG CGA GTG CAA AGT GG
tapasin_iso1_102	TTC GTT CCT TTG CCT GCG NNK GCA AAG TGG GCA TCA GGT
tapasin_iso1_125	CTT GAT GGA GCG TGG CTG NNK GTT TCC ATA AGC TCT CCG GT
tapasin_iso1_126	GAT GGA GCG TGG CTG ATG NNK TCC ATA AGC TCT CCG GTG C
tapasin_iso1_127	GGA GCG TGG CTG ATG GTT NNK ATA AGC TCT CCG GTG CTT TC
tapasin_iso1_128	AGC GTG GCT GAT GGT TTC CNN KAG CTC TCC GGT GCT TTC A
tapasin_iso1_129	GCG TGG CTG ATG GTT TCC ATA NNK TCT CCG GTG CTT TCA CTG
tapasin_iso1_130	TGG CTG ATG GTT TCC ATA AGC NNK CCG GTG CTT TCA CTG TCA
tapasin_iso1_131	GGC TGA TGG TTT CCA TAA GCT CTN NKG TGC TTT CAC TGT CAT CAC TG
tapasin_iso1_134	CCA TAA GCT CTC CGG TGC TTN NKC TGT CAT CAC TGC TCA GGC
tapasin_iso1_135	AGC TCT CCG GTG CTT TCA NNK TCA TCA CTG CTC AGG CCA
tapasin_iso1_136	CTC TCC GGT GCT TTC ACT GNN KTC ACT GCT CAG GCC ACA
tapasin_iso1_140	GCT TTC ACT GTC ATC ACT GCT CNN KCC ACA ACC TGA ACC CCA A
tapasin_iso1_144	CTG CTC AGG CCA CAA CCT NNK CCC CAA CAA GAA CCC GTA C
tapasin_iso1_157	CGT ACT GAT AAC AAT GGC GAC GNN KGT CCT TAC GGT CCT TAC GC
tapasin_iso1_164	GGT AGT CCT TAC GGT CCT TAC GNN KAC ACC AGC ACC CAG GG
tapasin_iso1_166	CCT TAC GGT CCT TAC GCA TAC ANN KGC ACC CAG GGT GCG
tapasin_iso1_167	CGG TCC TTA CGC ATA CAC CAN NKC CCA GGG TGC GAT TGG
tapasin_iso1_172	CCA GCA CCC AGG GTG CGA NNK GGA CAA GAC GCA CTC CTC
tapasin_iso1_180	ACA AGA CGC ACT CCT CGA CNN KAG CTT TGC GTA CAT GCC A
tapasin_iso1_182	CGC ACT CCT CGA CCT TAG CNN KGC GTA CAT GCC ACC AAC C
tapasin_iso1_206	CCA CCG TTC GGC CTT GAA NNK AGA CGA CAG CAC TTG GGG
tapasin_iso1_207	ACC GTT CGG CCT TGA ATG GNN KCG ACA GCA CTT GGG GAA
tapasin_iso1_209	TCG GCC TTG AAT GGA GAC GAN NKC ACT TGG GGA AGG GCC A
tapasin_iso1_210	GGC CTT GAA TGG AGA CGA CAG NNK TTG GGG AAG GGC CAT TTG
tapasin_iso1_211	TTG AAT GGA GAC GAC AGC ACN NKG GGA AGG GCC ATT TGC T
tapasin_iso1_212	ATG GAG ACG ACA GCA CTT GNN KAA GGG CCA TTT GCT GTT GG
tapasin_iso1_213	AGA CGA CAG CAC TTG GGG NNK GGC CAT TTG CTG TTG GC
tapasin_iso1_214	ACG ACA GCA CTT GGG GAA GNN KCA TTT GCT GTT GGC TGC TAC
tapasin_iso1_238	ACA AGA GGG AGC CGT AGC TNN KGC TGC GTG GGA TGA TGA
tapasin_iso1_241	GCC GTA GCT TTT GCT GCG NNK GAT GAT GAC GAA CCA TGG GG
tapasin_iso1_257	TGG ACT GGA AAC GGG ACT TTC NNK CTG CCA ACA GTA CAG CCC
tapasin_iso1_262	CTT TCT GGC TGC CAA CAG TAN NKC CCT TTC AAG AAG GAA CGT ATC
tapasin_iso1_268	AGT ACA GCC CTT TCA AGA AGG ANN KTA TCT GGC CAC CAT CCA TTT G
tapasin_iso1_269	CAG CCC TTT CAA GAA GGA ACG NNK CTG GCC ACC ATC CAT TTG C
tapasin_iso1_270	AGC CCT TTC AAG AAG GAA CGT ATN NKG CCA CCA TCC ATT TGC CT
tapasin_iso1_272	CAA GAA GGA ACG TAT CTG GCC NNK ATC CAT TTG CCT TAT CTG CAA G
tapasin_iso1_279	CCA CCA TCC ATT TGC CTT ATC TGN NKG GCC AGG TCA CAC TTG AA
tapasin_iso1_281	CCA TTT GCC TTA TCT GCA AGG CNN KGT CAC ACT TGA ACT CGC TG
tapasin_iso1_282	TGC CTT ATC TGC AAG GCC AGN NKA CAC TTG AAC TCG CTG TCT AT
tapasin_iso1_283	CTT ATC TGC AAG GCC AGG TCN NKC TTG AAC TCG CTG TCT ATA AAC C
tapasin_iso1_285	GCA AGG CCA GGT CAC ACT TNN KCT CGC TGT CTA TAA ACC GCC
tapasin_iso1_287	GGC CAG GTC ACA CTT GAA CTC NNK GTC TAT AAA CCG CCT AAG GTG A
tapasin_iso1_290	CAC ACT TGA ACT CGC TGT CTA TNN KCC GCC TAA GGT GAG TCT CA
tapasin_iso1_295	CTG TCT ATA AAC CGC CTA AGG TGN NKC TCA TGC CCG CAA CTT TG
tapasin_iso1_313	GGC GAA GCT CCA CCT GAA NNK CTC TGT TTG GTA TCC CAT TTC TAC
tapasin_iso1_318	CAC CTG AAT TGC TCT GTT TGG TAN NKC ATT TCT ACC CCA GTG GTG G
tapasin_iso1_321	TGC TCT GTT TGG TAT CCC ATT TCN NKC CCA GTG GTG GCC TTG
tapasin_iso1_322	GCT CTG TTT GGT ATC CCA TTT CTA CNN KAG TGG TGG CCT TGA AGT G
tapasin_iso1_323	GTT TGG TAT CCC ATT TCT ACC CCN NKG GTG GCC TTG AAG TGG AA
tapasin_iso1_324	TGG TAT CCC ATT TCT ACC CCA GTN NKG GCC TTG AAG TGG AAT GGG
tapasin_iso1_325	TCC CAT TTC TAC CCC AGT GGT NNK CTT GAA GTG GAA TGG GAA CTT AG
tapasin_iso1_326	TTC TAC CCC AGT GGT GGC NNK GAA GTG GAA TGG GAA CTT AGA GG
tapasin_iso1_327	TAC CCC AGT GGT GGC CTT NNK GTG GAA TGG GAA CTT AGA GGA G
tapasin_iso1_328	CCC CAG TGG TGG CCT TGA ANN KGA ATG GGA ACT TAG AGG AGG AC
tapasin_iso1_329	CAG TGG TGG CCT TGA AGT GNN KTG GGA ACT TAG AGG AGG ACC
tapasin_iso1_333	GCC TTG AAG TGG AAT GGG AAC TTN NKG GAG GAC CAG GTG GTC G
tapasin_iso1_33R	GTC TTC AAC AAA CCA ACA CTC GA
tapasin_iso1_34R	AGC GTC TTC AAC AAA CCA ACA
tapasin_iso1_35R	ACT AGC GTC TTC AAC AAA CCA A
tapasin_iso1_36R	ACC ACT AGC GTC TTC AAC AAA
tapasin_iso1_37R	TTT ACC ACT AGC GTC TTC AAC AA
tapasin_iso1_38R	CCC TTT ACC ACT AGC GTC TTC

tapasin_iso1_39R	CAG CCC TTT ACC ACT AGC GT
tapasin_iso1_88R	TGG AGC ACC TCT TGG GTA
tapasin_iso1_90R	CGG TGC TGG AGC ACC TCT
tapasin_iso1_92R	GCA ATG CGG TGC TGG AGC
tapasin_iso1_94R	CAT CTC GCA ATG CGG TGC
tapasin_iso1_95R	ACT CAT CTC GCA ATG CGG T
tapasin_iso1_96R	CCG ACT CAT CTC GCA ATG CG
tapasin_iso1_97R	GAA CCG ACT CAT CTC GCA A
tapasin_iso1_98R	AAC GAA CCG ACT CAT CTC GC
tapasin_iso1_99R	AGG AAC GAA CCG ACT CAT CT
tapasin_iso1_102R	CGC AGG CAA AGG AAC GAA
tapasin_iso1_125R	CAG CCA CGC TCC ATC AAG
tapasin_iso1_126R	CAT CAG CCA CGC TCC ATC
tapasin_iso1_127R	AAC CAT CAG CCA CGC TCC
tapasin_iso1_128R	GGA AAC CAT CAG CCA CGC T
tapasin_iso1_129R	TAT GGA AAC CAT CAG CCA CGC
tapasin_iso1_130R	GCT TAT GGA AAC CAT CAG CCA
tapasin_iso1_131R	AGA GCT TAT GGA AAC CAT CAG CC
tapasin_iso1_134R	AAG CAC CGG AGA GCT TAT GG
tapasin_iso1_135R	TGA AAG CAC CGG AGA GCT
tapasin_iso1_136R	CAG TGA AAG CAC CGG AGA G
tapasin_iso1_140R	GAG CAG TGA TGA CAG TGA AAG C
tapasin_iso1_144R	AGG TTG TGG CCT GAG CAG
tapasin_iso1_157R	CGT CGC CAT TGT TAT CAG TAC G
tapasin_iso1_164R	CGT AAG GAC CGT AAG GAC TAC C
tapasin_iso1_166R	TGT ATG CGT AAG GAC CGT AAG G
tapasin_iso1_167R	TGG TGT ATG CGT AAG GAC CG
tapasin_iso1_172R	TCG CAC CCT GGG TGC TGG
tapasin_iso1_180R	GTC GAG GAG TGC GTC TTG T
tapasin_iso1_182R	GCT AAG GTC GAG GAG TGC G
tapasin_iso1_206R	TTC AAG GCC GAA CGG TGG
tapasin_iso1_207R	CCA TTC AAG GCC GAA CGG T
tapasin_iso1_209R	TCG TCT CCA TTC AAG GCC GA
tapasin_iso1_210R	CTG TCG TCT CCA TTC AAG GCC
tapasin_iso1_211R	GTG CTG TCG TCT CCA TTC AA
tapasin_iso1_212R	CAA GTG CTG TCG TCT CCA T
tapasin_iso1_213R	CCC CAA GTG CTG TCG TCT
tapasin_iso1_214R	CTT CCC CAA GTG CTG TCG T
tapasin_iso1_238R	AGC TAC GGC TCC CTC TTG T
tapasin_iso1_241R	CGC AGC AAA AGC TAC GGC
tapasin_iso1_257R	GAA AGT CCC GTT TCC AGT CCA
tapasin_iso1_262R	TAC TGT TGG CAG CCA GAA AG
tapasin_iso1_268R	TCC TTC TTG AAA GGG CTG TAC T
tapasin_iso1_269R	CGT TCC TTC TTG AAA GGG CTG
tapasin_iso1_270R	ATA CGT TCC TTC TTG AAA GGG CT
tapasin_iso1_272R	GGC CAG ATA CGT TCC TTC TTG
tapasin_iso1_279R	CAG ATA AGG CAA ATG GAT GGT GG
tapasin_iso1_281R	GCC TTG CAG ATA AGG CAA ATG G
tapasin_iso1_282R	CTG GCC TTG CAG ATA AGG CA
tapasin_iso1_283R	GAC CTG GCC TTG CAG ATA AG
tapasin_iso1_285R	AAG TGT GAC CTG GCC TTG C
tapasin_iso1_287R	GAG TTC AAG TGT GAC CTG GCC
tapasin_iso1_290R	ATA GAC AGC GAG TTC AAG TGT G
tapasin_iso1_295R	CAC CTT AGG CGG TTT ATA GAC AG
tapasin_iso1_313R	TTC AGG TGG AGC TTC GCC
tapasin_iso1_318R	TAC CAA ACA GAG CAA TTC AGG TG
tapasin_iso1_321R	GAA ATG GGA TAC CAA ACA GAG CA
tapasin_iso1_322R	GTA GAA ATG GGA TAC CAA ACA GAG C
tapasin_iso1_323R	GGG GTA GAA ATG GGA TAC CAA AC
tapasin_iso1_324R	ACT GGG GTA GAA ATG GGA TAC CA
tapasin_iso1_325R	ACC ACT GGG GTA GAA ATG GGA
tapasin_iso1_326R	GCC ACC ACT GGG GTA GAA
tapasin_iso1_327R	AAG GCC ACC ACT GGG GTA
tapasin_iso1_328R	TTC AAG GCC ACC ACT GGG G
tapasin_iso1_329R	CAC TTC AAG GCC ACC ACT G
tapasin_iso1_333R	AAG TTC CCA TTC CAC TTC AAG GC

tapasin_iso1_342	GAC CAG GTG GTC GAT CTC AGN NKG CTG AAG GGC AGC GAT
tapasin_iso1_343	CCA GGT GGT CGA TCT CAG AAA NNK GAA GGG CAG CGA TGG C
tapasin_iso1_344	GGT GGT CGA TCT CAG AAA GCT NNK GGG CAG CGA TGG CTC
tapasin_iso1_345	TGG TCG ATC TCA GAA AGC TGA ANN KCA GCG ATG GCT CTC CG
tapasin_iso1_346	TCG ATC TCA GAA AGC TGA AGG GNN KCG ATG GCT CTC CGC TC
tapasin_iso1_347	CTC AGA AAG CTG AAG GGC AGN NKT GGC TCT CCG CTC TCA G
tapasin_iso1_348	AAA GCT GAA GGG CAG CGA NNK CTC TCC GCT CTC AGG CA
tapasin_iso1_349	GCT GAA GGG CAG CGA TGG NNK TCC GCT CTC AGG CAC C
tapasin_iso1_350	GAA GGG CAG CGA TGG CTC NNK GCT CTC AGG CAC CAC TC
tapasin_iso1_351	GGG CAG CGA TGG CTC TCC NNK CTC AGG CAC CAC TCA GAC
tapasin_iso1_352	CAG CGA TGG CTC TCC GCT NNK AGG CAC CAC TCA GAC GG
tapasin_iso1_353	GCG ATG GCT CTC CGC TCT CNN KCA CCA CTC AGA CGG ATC TG
tapasin_iso1_354	TGG CTC TCC GCT CTC AGG NNK CAC TCA GAC GGA TCT GTA TCA C
tapasin_iso1_355	CTC TCC GCT CTC AGG CAC NNK TCA GAC GGA TCT GTA TCA CTG T
tapasin_iso1_356	TCC GCT CTC AGG CAC CAC NNK GAC GGA TCT GTA TCA CTG TCA
tapasin_iso1_357	CGC TCT CAG GCA CCA CTC ANN KGG ATC TGT ATC ACT GTC AGG G
tapasin_iso1_358	TCT CAG GCA CCA CTC AGA CNN KTC TGT ATC ACT GTC AGG GCA
tapasin_iso1_359	AGG CAC CAC TCA GAC GGA NNK GTA TCA CTG TCA GGG CAT TTG
tapasin_iso1_360	GGC ACC ACT CAG ACG GAT CTN NKT CAC TGT CAG GGC ATT TGC
tapasin_iso1_361	CAC CAC TCA GAC GGA TCT GTA NNK CTG TCA GGG CAT TTG CAG C
tapasin_iso1_362	ACC ACT CAG ACG GAT CTG TAT CAN NKT CAG GGC ATT TGC AGC C
tapasin_iso1_363	ACT CAG ACG GAT CTG TAT CAC TGN NKG GGC ATT TGC AGC CAC C
tapasin_iso1_365	CGG ATC TGT ATC ACT GTC AGG GNN KTT GCA GCC ACC GCC
tapasin_iso1_367	TGT ATC ACT GTC AGG GCA TTT GNN KCC ACC GCC AGT TAC CAC
tapasin_iso1_381	AGC AGC ATG GAG CTC GGT ATN NKT GTC GCA TTC ACC ACC C
tapasin_iso1_382	GCA TGG AGC TCG GTA TGC GNN KCG CAT TCA CCA CCC TAG C
tapasin_iso1_386	GGT ATG CGT GTC GCA TTC ACN NKC CTA GCC TTC CGG CGT
tapasin_iso1_387	TGC GTG TCG CAT TCA CCA CNN KAG CCT TCC GGC GTC A
tapasin_iso1_390	CGC ATT CAC CAC CCT AGC CTT NNK GCG TCA GGA AGA TCA GCG
tapasin_iso1_30	TGC CGT AAT CGA GTG TTG GTT TNN KGA AGA CGC TAG TGG TAA AGG G
tapasin_iso1_31	CCG TAA TCG AGT GTT GGT TTG TTN NKG ACG CTA GTG GTA AAG GGC
tapasin_iso1_32	TAA TCG AGT GTT GGT TTG TTG AAN NKG CTA GTG GTA AAG GGC TGG
tapasin_iso1_40	TAG TGG TAA AGG GCT GGC TNN KCG GCC AGG CGC AC
tapasin_iso1_297	AAC CGC CTA AGG TGA GTC TCN NKC CCG CAA CTT TGG CTA GG
tapasin_iso1_314	GGC GAA GCT CCA CCT GAA TTG NNK TGT TTG GTA TCC CAT TTC TAC CC
tapasin_iso1_316	GCT CCA CCT GAA TTG CTC TGT NNK GTA TCC CAT TTC TAC CCC AGT G
tapasin_iso1_342R	CTG AGA TCG ACC ACC TGG TC
tapasin_iso1_343R	TTT CTG AGA TCG ACC ACC TGG
tapasin_iso1_344R	AGC TTT CTG AGA TCG ACC ACC
tapasin_iso1_345R	TTC AGC TTT CTG AGA TCG ACC A
tapasin_iso1_346R	CCC TTC AGC TTT CTG AGA TCG A
tapasin_iso1_347R	CTG CCC TTC AGC TTT CTG AG
tapasin_iso1_348R	TCG CTG CCC TTC AGC TTT
tapasin_iso1_349R	CCA TCG CTG CCC TTC AGC
tapasin_iso1_350R	GAG CCA TCG CTG CCC TTC
tapasin_iso1_351R	GGA GAG CCA TCG CTG CCC
tapasin_iso1_352R	AGC GGA GAG CCA TCG CTG
tapasin_iso1_353R	GAG AGC GGA GAG CCA TCG C
tapasin_iso1_354R	CCT GAG AGC GGA GAG CCA
tapasin_iso1_355R	GTG CCT GAG AGC GGA GAG
tapasin_iso1_356R	GTG GTG CCT GAG AGC GGA
tapasin_iso1_357R	TGA GTG GTG CCT GAG AGC G
tapasin_iso1_358R	GTC TGA GTG GTG CCT GAG A
tapasin_iso1_359R	TCC GTC TGA GTG GTG CCT
tapasin_iso1_360R	AGA TCC GTC TGA GTG GTG CC
tapasin_iso1_361R	TAC AGA TCC GTC TGA GTG GTG
tapasin_iso1_362R	TGA TAC AGA TCC GTC TGA GTG GT
tapasin_iso1_363R	CAG TGA TAC AGA TCC GTC TGA GT
tapasin_iso1_365R	CCC TGA CAG TGA TAC AGA TCC G
tapasin_iso1_367R	CAA ATG CCC TGA CAG TGA TAC A
tapasin_iso1_381R	ATA CCG AGC TCC ATG CTG CT
tapasin_iso1_382R	CGC ATA CCG AGC TCC ATG C
tapasin_iso1_386R	GTG AAT GCG ACA CGC ATA CC
tapasin_iso1_387R	GTG GTG AAT GCG ACA CGC A
tapasin_iso1_390R	AAG GCT AGG GTG GTG AAT GCG
tapasin_iso1_30R	AAA CCA ACA CTC GAT TAC GGC A

tapasin_iso1_31R	AAC AAA CCA ACA CTC GAT TAC GG
tapasin_iso1_32R	TTC AAC AAA CCA ACA CTC GAT TA
tapasin_iso1_40R	AGC CAG CCC TTT ACC ACT A
tapasin_iso1_297R	GAG ACT CAC CTT AGG CGG TT
tapasin_iso1_314R	CAA TTC AGG TGG AGC TTC GCC
tapasin_iso1_316R	ACA GAG CAA TTC AGG TGG AGC
TAPBPR_E102A_for	GGA AGG AGG TGA CCT GTG CTA TCT CCC GCT ACT TTC
TAPBPR_M122A_for	CAG CAG CTT GGT TCG CTG CCA ACG TGC AGG TCT C
TAPBPR_G212A_for	GAC TGC AGC ACA AGG CCA GGG GTC AGT TGG TG
TAPBPR_I261A_for	GAC GAG GGG ACC TAC GCT TGC CAG ATC ACC ACC
TAPBPR_S333A_for	GTG CCT CCT TCT CCG CCC TCA GGC AAA GCG TG
TAPBPR_E102A_rev	GAA AGT AGC GGG AGA TAG CAC AGG TCA CCT CCT TCC
TAPBPR_M122A_rev	GAG ACC TGC ACG TTG GCA GCG AAC CAA GCT GCT G
TAPBPR_G212A_rev	CAC CAA CTG ACC CCT GGC CTT GTG CTG CAG TC
TAPBPR_I261A_rev	GGT GGT GAT CTG GCA AGC GTA GGT CCC CTC GTC
TAPBPR_S333A_rev	CAC GCT TTG CCT GAG GGC GGA GAA GGA GGC AC
TAPBPR-delta24-36-R	CAC AAG GGA GGC CCT TGC GTC CTT CAC CAG GAA ACA GTC
TAPBPR-delta24-26-F	GAC TGT TTC CTG GTG AAG GAC GCA AGG GCC TCC CTT GTG
TAPBPR-dALAS-for	GACGGTGCGCACCGTGGAagtgaggacagggcaaggg
TAPBPR-dALAS-rev	cccttgccctgtcctcactTCCACGGTGCGCACCGTC
Tapasin-TAPBPR22-25 For	CAC CGT GGA GCT CTC GCC AGC AGT GAG GAC AAA CGG CCA GGC GCA CTT C
Tapasin-TAPBPR22-25-Rev	TGGCGAGAGCTCCACGGTGCGCACCGTCCTTAACAAACCAACACTCGATTACGG
Tapasin-d2steps-F	CGA GTG TTG GTT TGT TGA AGA CGG TCT GGC TAA ACG GCC AGG
Tapasin-d2steps-R	CCT GGC CGT TTA GCC AGA CCG TCT TCA ACA AAC CAA CAC TCG
Tapasin-G15L-F	GTT GAA GAC GCT AGT CTG AAA GGG CTG GCT AAA CGG C
Tapasin-G15L-R	GCC GTT TAG CCA GCC CTT TCA GAC TAG CGT CTT CAA C
Tapasin-G15E-F	GTT GAA GAC GCT AGT GAA AAA GGG CTG GCT AAA CGG CCA G
Tapasin-G15E-R	CTG GCC GTT TAG CCA GCC CTT TTT CAC TAG CGT CTT CAA C
Tapasin-L18G-F	CTA GTG GTA AAG GGG GCG CTA AAC GGC CAG G
Tapasin-L18G-R	CCT GGC CGT TTA GCG CCC CCT TTA CCA CTA G
Tapasin-L18E-F	GCT AGT GGT AAA GGG GAA GCT AAA CGG CCA GGC G
Tapasin-L18E-R	CGC CTG GCC GTT TAG CTT CCC CTT TAC CAC TAG C
Tapasin-L18K-F	CGC TAG TGG TAA AGG GAA AGC TAA ACG GCC AGG CG
Tapasin-L18K-R	CGC CTG GCC GTT TAG CTT TCC CTT TAC CAC TAG CG
Tapasin-D2 to d3steps F	CGA GTG TTG GTT TGT TGA AGG TGC TAA ACG GCC AGG CG
Tapasin-D2 to d3steps R	CGC CTG GCC GTT TAG CAC CTT CAA CAA ACC AAC ACT CG
Illumina_tapasin_5F	TCTTTCCCTACACGACGCTCTTCCGATCTGCCGTAATCGAGTGTTGGT
Illumina_tapasin_235R	GTGACTGGAGTTCAGACGTGTGCTCTTCCGATCTAAGGAACGAACCGACTCATC
Illumina_tapasin_183F	TCTTTCCCTACACGACGCTCTTCCGATCTGTACCCAAGAGGTGCTCCA
Illumina_tapasin_405R	GTGACTGGAGTTCAGACGTGTGCTCTTCCGATCTCGCCATTGTTATCAGTACGG
Illumina_tapasin_386F	TCTTTCCCTACACGACGCTCTTCCGATCTCCGTACTGATAACAATGGCG
Illumina_tapasin_602R	GTGACTGGAGTTCAGACGTGTGCTCTTCCGATCTGTAGCAGCCAACAGCAAATG
Illumina_tapasin_630F	TCTTTCCCTACACGACGCTCTTCCGATCTAGCACAAGAGGGAGCCGTA
Illumina_tapasin_876R	GTGACTGGAGTTCAGACGTGTGCTCTTCCGATCTTCAGGTGGAGCTTCGCCA
Illumina_tapasin_858F	TCTTTCCCTACACGACGCTCTTCCGATCTGGCGAAGCTCCACCTGAA
Illumina_tapasin_1040R	GTGACTGGAGTTCAGACGTGTGCTCTTCCGATCTGCAAATGCCCTGACAGTGA
Illumina_tapasin_951F	TCTTTCCCTACACGACGCTCTTCCGATCTGGTCGATCTCAGAAAGCTG
Illumina_tapasin_1135R	GTGACTGGAGTTCAGACGTGTGCTCTTCCGATCTTACCTCCGCTGATCTTCCT
Illumina_TAPBPR_273_for	TCTTTCCCTACACGACGCTCTTCCGATcttgctccatgctgactgca
Illumina_TAPBPR_483_rev	GTGACTGGAGTTCAGACGTGTGCTCTTCCGATCTCAGTGGCAAGTTCAGCGTTG
Illumina_TAPBPR_466_for	TCTTTCCCTACACGACGCTCTTCCGATCTacgctgaacttgccactgag
Illumina_TAPBPR_671_rev	GTGACTGGAGTTCAGACGTGTGCTCTTCCGATCTGTCCAGCTGTACACCAACTG
Illumina_TAPBPR_649_for	TCTTTCCCTACACGACGCTCTTCCGATCTggtcagttggtgtacagctg
Illumina_TAPBPR_920_rev	GTGACTGGAGTTCAGACGTGTGCTCTTCCGATCTCCAGCAATGTCGCAGATGAG
Illumina_TAPBPR_875_for	TCTTTCCCTACACGACGCTCTTCCGATCtggcaaacgaagctctgctg
Illumina_TAPBPR_1148_rev	GTGACTGGAGTTCAGACGTGTGCTCTTCCGATCTGGGACAACCTGGGTGCTG
Illumina_Start_Adaptamer	AATGATACGGCGACCACCGAGATCTACACTCTTTCCCTACACGACGCTCTTCCGATCT
Illumina_Index_1_Adaptamer	CAAGCAGAAGACGGCATACGAGATCGTGATGTGACTGGAGTTCAGACGTGTGCTCTTC
Illumina_Index_2_Adaptamer	CAAGCAGAAGACGGCATACGAGATACATCGGTGACTGGAGTTCAGACGTGTGCTCTTC
Illumina_Index_3_Adaptamer	CAAGCAGAAGACGGCATACGAGATGCCTAAGTGACTGGAGTTCAGACGTGTGCTCTTC
Illumina_Index_4_Adaptamer	CAAGCAGAAGACGGCATACGAGATTGGTCAGTGACTGGAGTTCAGACGTGTGCTCTTC
Illumina_Index_5_Adaptamer	CAAGCAGAAGACGGCATACGAGATCACTGTGTGACTGGAGTTCAGACGTGTGCTCTTC
Illumina_Index_6_Adaptamer	CAAGCAGAAGACGGCATACGAGATATTGGCGTGACTGGAGTTCAGACGTGTGCTCTTC
Illumina_Index_7_Adaptamer	CAAGCAGAAGACGGCATACGAGATGATCTGGTGACTGGAGTTCAGACGTGTGCTCTTC
Illumina_Index_8_Adaptamer	CAAGCAGAAGACGGCATACGAGATTCAAGTGTGACTGGAGTTCAGACGTGTGCTCTTC
Illumina_Index_9_Adaptamer	CAAGCAGAAGACGGCATACGAGATCTGATCGTGACTGGAGTTCAGACGTGTGCTCTTC

Illumina_Index_10_Adaptamer	CAAGCAGAAGACGGCATACGAGATGTAGCCGTGACTGGAGTTCAGACGTGTGCTCTTC
Illumina_Index_11_Adaptamer	CAAGCAGAAGACGGCATACGAGATTACAAGGTGACTGGAGTTCAGACGTGTGCTCTTC
Illumina_Index_12_Adaptamer	CAAGCAGAAGACGGCATACGAGATATCAGTGTGACTGGAGTTCAGACGTGTGCTCTTC
Illumina_Index_13_Adaptamer	CAAGCAGAAGACGGCATACGAGATGCTCATGTGACTGGAGTTCAGACGTGTGCTCTTC
Illumina_Index_14_Adaptamer	CAAGCAGAAGACGGCATACGAGATAGGAATGTGACTGGAGTTCAGACGTGTGCTCTTC
Illumina_Index_15_Adaptamer	CAAGCAGAAGACGGCATACGAGATCTTTTGGTGACTGGAGTTCAGACGTGTGCTCTTC
Illumina_Index_16_Adaptamer	CAAGCAGAAGACGGCATACGAGATTAGTTGGTGACTGGAGTTCAGACGTGTGCTCTTC
Illumina_Index_17_Adaptamer	CAAGCAGAAGACGGCATACGAGATCCGGTGGTGACTGGAGTTCAGACGTGTGCTCTTC
Illumina_Index_18_Adaptamer	CAAGCAGAAGACGGCATACGAGATTTGACTGTGACTGGAGTTCAGACGTGTGCTCTTC
Illumina_Index_19_Adaptamer	CAAGCAGAAGACGGCATACGAGATGGAACTGTGACTGGAGTTCAGACGTGTGCTCTTC
Illumina_Index_20_Adaptamer	CAAGCAGAAGACGGCATACGAGATTGACATGTGACTGGAGTTCAGACGTGTGCTCTTC
Illumina_Index_21_Adaptamer	CAAGCAGAAGACGGCATACGAGATGGACGGGTGACTGGAGTTCAGACGTGTGCTCTTC
Illumina_Index_22_Adaptamer	CAAGCAGAAGACGGCATACGAGATCTCTACGTGACTGGAGTTCAGACGTGTGCTCTTC
Illumina_Index_23_Adaptamer	CAAGCAGAAGACGGCATACGAGATGCGGACGTGACTGGAGTTCAGACGTGTGCTCTTC
Illumina_Index_24_Adaptamer	CAAGCAGAAGACGGCATACGAGATTTTCACGTGACTGGAGTTCAGACGTGTGCTCTTC
Illumina_Index_25_Adaptamer	CAAGCAGAAGACGGCATACGAGATGGCCACGTGACTGGAGTTCAGACGTGTGCTCTTC
Illumina_Index_26_Adaptamer	CAAGCAGAAGACGGCATACGAGATCGAAACGTGACTGGAGTTCAGACGTGTGCTCTTC
Illumina_Index_27_Adaptamer	CAAGCAGAAGACGGCATACGAGATCGTACGGTGACTGGAGTTCAGACGTGTGCTCTTC
Illumina_Index_28_Adaptamer	CAAGCAGAAGACGGCATACGAGATCCACTCGTGACTGGAGTTCAGACGTGTGCTCTTC
Illumina_Index_29_Adaptamer	CAAGCAGAAGACGGCATACGAGATGCTACCGTGACTGGAGTTCAGACGTGTGCTCTTC
Illumina_Index_30_Adaptamer	CAAGCAGAAGACGGCATACGAGATAAGCTAGTGACTGGAGTTCAGACGTGTGCTCTTC
pCEP4-MCS-for	GAT CTC TAG AAG CTG GGT ACC
pCEP4-MCS-rev	CAA TGT ATC TTA TCA TGT CTG GAT CC
TAPBPR_L30K_for	GCGCACCGTGGAGCTAAAGCCAGCAGTGAGGAC
TAPBPR_L30K_rev	GTCCTCACTGCTGGCTTTAGCTCCACGGTGCGC
TAPBPR_D35F_for	CTCGCCAGCAGTGAGTTCAGGGCAAGGGCCTC
TAPBPR_D35F_rev	GAGGCCCTTGCCCTGAACTCACTGCTGGCGAG
TAPBPR_D35R_for	CTCGCCAGCAGTGAGCGCAGGGCAAGGGCCTC
TAPBPR_D35R_rev	GAGGCCCTTGCCCTGCGCTCACTGCTGGCGAG
TAPBPR_D35N_for	CTCGCCAGCAGTGAGAACAGGGCAAGGGCCTC
TAPBPR_D35N_rev	GAGGCCCTTGCCCTGTTCTCACTGCTGGCGAG
TAPBPR_A25L_for	CTGGTGAAGGACGGTCTGCACCGTGGAGCTCTC
TAPBPR_A25L_rev	GAGAGCTCCACGGTGCAGACCGTCCTTCACCAG
TAPBPR_H26F_for	GTGAAGGACGGTGCGTTCCGTGGAGCTCTCGC
TAPBPR_H26F_rev	GCGAGAGCTCCACGGAACGCACCGTCCTTCAC
TAPBPR_R27E_for	GAAGGACGGTGCGCACGAAGGAGCTCTCGCCAGC
TAPBPR_R27E_rev	GCTGGCGAGAGCTCCTTCGTGCGCACCGTCCTTC
TAPBPR_A29L_for	GTGCGCACCGTGGACTTCTCGCCAGCAGTG
TAPBPR_A29L_rev	CACTGCTGGCGAGAAGTCCACGGTGCGCAC
TAPBPR_A31W_for	CACCGTGGAGCTCTCTGGAGCAGTGAGGACAGG
TAPBPR_A31W_rev	CCTGTCCTCACTGCTCCAGAGAGCTCCACGGTG
TAPBPR_S32L_for	GTGGAGCTCTCGCCCTCAGTGAGGACAGGGC
TAPBPR_S32L_rev	GCCCTGTCCTCACTGAGGGCGAGAGCTCCAC
TAPBPR_S32Y_for	GTGGAGCTCTCGCCTACAGTGAGGACAGGGC
TAPBPR_S32Y_rev	GCCCTGTCCTCACTGTAGGCGAGAGCTCCAC
TAPBPR_R27W_for	GAAGGACGGTGCGCACTGGGGAGCTCTCGCCAGC
TAPBPR_R27W_rev	GCTGGCGAGAGCTCCCCAGTGCGCACCGTCCTTC
TAPBPR_E34I_for	GCTCTCGCCAGCAGTATCGACAGGGCAAGGGC
TAPBPR_E34I_rev	GCCCTTGCCCTGTCGATACTGCTGGCGAGAGC
TAPBPR_E34Y_for	GCTCTCGCCAGCAGTTATGACAGGGCAAGGGC
TAPBPR_E34Y_rev	GCCCTTGCCCTGTCATAACTGCTGGCGAGAGC
TAPBPR_D35G_for	CTCGCCAGCAGTGAGGGCAGGGCAAGGGCCTC
TAPBPR_D35G_rev	GAGGCCCTTGCCCTGCCCTCACTGCTGGCGAG
TAPBPR-R405_nostop_rev	TCTCCGCTCTGGTGGGACAA
Tapasin-S386-TAPBPR-fusion	TTGTCCCACCAGAGCGGAGATCAGGGCCCTCCCTTGAG
Tapasin-T484-rev	GTGGATGTCTCGAGTCATTAAGTAGCATGCTCAAAGAGTCC
