# Supplementary material for: TAPBPR promotes antigen loading on MHC-I molecules using a peptide trap
Source: Nat Commun. 2021 May 26;12:3174. doi: 10.1038/s41467-021-23225-6 (PMC8154891; doi:10.1038/s41467-021-23225-6)
Supplement: Supplementary file 3 — Reporting summary [file 41467_2021_23225_MOESM3_ESM.pdf]

nature research

Corresponding author(s): Nikolaos G. Sgourakis

Last updated by author(s): March 23, 2021

Reporting Summary

Nature Research wishes to improve the reproducibility of the work that we publish. This form provides structure for consistency and transparency in reporting. For further information on Nature Research policies, see our Editorial Policies and the Editorial Policy Checklist.

Statistics

For all statistical analyses, confirm that the following items are present in the figure legend, table legend, main text, or Methods section.

n/a	Confirmed	
☐	☒	The exact sample size (*n*) for each experimental group/condition, given as a discrete number and unit of measurement
☐	☒	A statement on whether measurements were taken from distinct samples or whether the same sample was measured repeatedly
☒	☐	The statistical test(s) used AND whether they are one- or two-sided<br>*Only common tests should be described solely by name; describe more complex techniques in the Methods section.*
☒	☐	A description of all covariates tested
☒	☐	A description of any assumptions or corrections, such as tests of normality and adjustment for multiple comparisons
☐	☒	A full description of the statistical parameters including central tendency (e.g. means) or other basic estimates (e.g. regression coefficient) AND variation (e.g. standard deviation) or associated estimates of uncertainty (e.g. confidence intervals)
☒	☐	For null hypothesis testing, the test statistic (e.g. *F*, *t*, *r*) with confidence intervals, effect sizes, degrees of freedom and *P* value noted<br>*Give P values as exact values whenever suitable.*
☒	☐	For Bayesian analysis, information on the choice of priors and Markov chain Monte Carlo settings
☒	☐	For hierarchical and complex designs, identification of the appropriate level for tests and full reporting of outcomes
☒	☐	Estimates of effect sizes (e.g. Cohen's *d*, Pearson's *r*), indicating how they were calculated

Our web collection on statistics for biologists contains articles on many of the points above.

Software and code

Policy information about availability of computer code

Data collection	NMR: Topspin 3.5pl7 (Bruker); Illumina Sequencing: NovaSeq 6000 or HiSeq 4000 ; Deep Mutagenesis BD FACSAria II; Flow Cyometry: BD LSRII
Data analysis	Disulfide constructs were designed using Disulfide by Design v2. Fitting of DSF and FP data was performed using GraphPad Prism v7. Rosetta: Rosetta v2018.17 was used. MD: GROMACS version 2019.2 was used. Analysis of NMR data was performed using NMRPipe (Version 10.9 Rev 2020.119.13.27, Frank DeLaglio, NIST) and NMRFAM SPARKY (Version 1.3, Tom Goddard and Tom Ferrin). All these softwares are available in the public domain, and all relevant parameters used to analyze the data are outlined in detail in the Methods section. Origin v7 (OriginLab Corporation) Sequencing data were analyzed with Enrich (Fowler, et al., 2011) and commands are outlined in the GEO data deposition. Flow cytometry FCS Express v6 (De Novo Software). ITC data was analyzed using Origin v7.

For manuscripts utilizing custom algorithms or software that are central to the research but not yet described in published literature, software must be made available to editors and reviewers. We strongly encourage code deposition in a community repository (e.g. GitHub). See the Nature Research guidelines for submitting code & software for further information.

Data

Policy information about availability of data

All manuscripts must include a data availability statement. This statement should provide the following information, where applicable:

- Accession codes, unique identifiers, or web links for publicly available datasets
- A list of figures that have associated raw data
- A description of any restrictions on data availability

Plasmids are deposited with Addgene (ID numbers 141308-9 and 153471-8). All Illumina sequencing data is deposited with GEO under series accession numbers GSE147137, GSE126206, GSE159247 and GSE118568. NMR assignments have been deposited into the Biological Magnetic Resonance Data Bank (http://www.bmrb.wisc.edu) under accession numbers 28107 and 28108. Previously solved structures were obtained from the Protein Data Bank (https://www.rcsb.org/).

Field-specific reporting

Please select the one below that is the best fit for your research. If you are not sure, read the appropriate sections before making your selection.

- [x] Life sciences
- [ ] Behavioural & social sciences
- [ ] Ecological, evolutionary & environmental sciences

For a reference copy of the document with all sections, see nature.com/documents/nr-reporting-summary-flat.pdf

Life sciences study design

All studies must disclose on these points even when the disclosure is negative.

Sample size

Sample size is not applicable to NMR measurements described in the manuscript since our NMR experimental results do not report and do not depend on quantitative values. Deep mutational scans were independently replicated twice, according to the standards of the field. The numbers of replicates for biochemical activity assays are indicated in figure legends and are standard for the field. We used at least 2 technical replicates to endure reproducibility of our results.

Data exclusions

No data were excluded in this study.

Replication

All deep mutational scans were replicated at least twice.The number of replications are provided in the manuscript. The replicates used the same plasmid or naive yeast libraries, but were otherwise completely independent and are considered biological replicates. Activity assays of targeted mutants were replicated using independently transfected cells. None of the processed data are from technical replicates; all processed mean values and deviations/errors represent biological replicates. Data replication does not apply to NMR measurements described in this manuscript. Standard referencing of chemical shifts is applied to ensure consistency and reproducibility. All attempts at replication were successful.

Randomization

Randomization does not apply to measurements described in this manuscript since there are no head-head comparisons between cohorts.

Blinding

Blinding is not relevant to this study.

Reporting for specific materials, systems and methods

We require information from authors about some types of materials, experimental systems and methods used in many studies. Here, indicate whether each material, system or method listed is relevant to your study. If you are not sure if a list item applies to your research, read the appropriate section before selecting a response.

Materials & experimental systems

n/a	Involved in the study
[ ]	[x] Antibodies
[ ]	[x] Eukaryotic cell lines
[x]	[ ] Palaeontology and archaeology
[x]	[ ] Animals and other organisms
[x]	[ ] Human research participants
[x]	[ ] Clinical data
[x]	[ ] Dual use research of concern

Methods

n/a	Involved in the study
[x]	[ ] ChIP-seq
[ ]	[x] Flow cytometry
[x]	[ ] MRI-based neuroimaging

Antibodies

Antibodies used

BioLegend 343306 (anti-human HLA-A2 PE, clone BB7.2); Sigma A9469 (anti-FLAG AP, clone M2); Invitrogen PA1 027A (rabbit anti-cyclophilin B); Jackson ImmunoResearch Laboratories 111-035-003 (goat anti-rabbit HRP); Immunology Consultants Laboratory CMYC-45F (FITC-conjugated chicken anti-c-myc)

Validation

All antibodies are commercially available and bind common epitope tags. All antibodies were shown within the lab to be specific for cells transfected/transformed with relevant tagged proteins.

Eukaryotic cell lines

Policy information about cell lines

Cell line source(s)

Expi293F cells were purchased from ThermoFisher (cat. no. A14527)

Authentication

The cell line was not authenticated.

Mycoplasma contamination

Frozen stocks of cells were confirmed to be negative for mycoplasma by PCR.

Commonly misidentified lines (See ICLAC register)

No commonly misidentified cell lines were used in this study.

Flow Cytometry

Plots

Confirm that:

- [x] The axis labels state the marker and fluorochrome used (e.g. CD4-FITC).
- [x] The axis scales are clearly visible. Include numbers along axes only for bottom left plot of group (a 'group' is an analysis of identical markers).
- [x] All plots are contour plots with outliers or pseudocolor plots.
- [x] A numerical value for number of cells or percentage (with statistics) is provided.

Methodology

Sample preparation	Transfected Expi293F cells or transformed yeast were analyzed by flow cytometry as described in the Methods.
Instrument	Sorting was on a BD FACS Aria II. Expi293F cells were analyzed on a BD LSRII. Yeast were analyzed on a BD Accuri C6.
Software	Flow cytometry data were analyzed on the BD Accuri C6 instrument software or with FCS Express v5 or v6.
Cell population abundance	Sorted populations for deep mutagenesis were analyzed by Illumina sequencing to determine the enrichment of particular sequence variants. The enrichment ratios and raw deep sequencing data are deposited in GEO.
Gating strategy	The gating strategy is provided in Supplementary Fig. 11.

- [x] Tick this box to confirm that a figure exemplifying the gating strategy is provided in the Supplementary Information.
